# Integrative Pharmacokinetic and Metabolomic Profiling of *Polygonum capitatum* Extract Reveals Renoprotective Mechanisms in a Rat Model of Acute Pyelonephritis

**DOI:** 10.3390/ijms27104399

**Published:** 2026-05-14

**Authors:** Xiaoliang Zhao, Zhaoyue Yuan, An Liu, Wenguang Jing, Weifeng Yang, Yue Jiao, Yang Liu, Chang Gao, Runzi Bai, Zhiguo Wang, Tao Li

**Affiliations:** 1 Experimental Research Center, China Academy of Chinese Medical Sciences, Beijing 100700, China; zhaoxiaoliang@merc.ac.cn (X.Z.); zyueyuan1118@163.com (Z.Y.);; 2 Institute of Chinese Materia Medica, China Academy of Chinese Medical Sciences, Beijing 100700, China; 3 Institute for Control of Traditional Chinese Medicine and Ethnic Medicine, National Institutes for Food and Drug Control, Beijing 102629, China

**Keywords:** acute pyelonephritis, urinary tract infection, *Polygonum capitatum*, pharmacokinetics, metabolomics

## Abstract

*Polygonum capitatum* (PC) is an ethnomedicine with reported antibacterial and anti-inflammatory activities and has been clinically used in urinary tract infection (UTI)-related disorders. However, its in vivo exposure characteristics and metabolically associated therapeutic mechanisms in acute pyelonephritis (AP) remain insufficiently understood. To address this issue, this study aimed to evaluate the therapeutic effects of PC in an *Escherichia coli* (*E. coli*)-induced rat model of AP and to explore constituents and metabolic pathways associated with its activity. Different PC extracts were screened for antibacterial and anti-inflammatory activities, and the 70% ethanol extract was selected for further study. Seven major compounds were quantified by HPLC. In AP rats, the pharmacokinetic profiles of these compounds in plasma and the renal cortex were analyzed by microdialysis-coupled HPLC-MS/MS. Pharmacodynamic evaluation included urinary bacterial load, urinalysis, renal function, inflammatory cytokines, and renal histopathology. Exploratory PK–PD analysis, untargeted renal metabolomics, and targeted metabolomics of the tryptophan–kynurenine (Trp–Kyn) pathway were also performed. The 70% ethanol extract of PC exhibited the strongest antibacterial and anti-inflammatory activities. The total content of seven active compounds was 3.85%, with gallic acid being the predominant compound (3.42%). Pharmacokinetic analysis revealed that gallic acid, protocatechuic acid, methyl gallate, and quercitrin achieved relatively high systemic exposure and renal distribution. In AP rats, the pharmacokinetic profiles of several compounds were altered, with increased plasma exposure of protocatechuic acid, vanillic acid, ethyl gallate, and syringic acid, while protocatechuic acid also showed higher exposure in the renal cortex. PC treatment reduced urinary bacterial load, improved renal function and urinalysis parameters, alleviated histopathological injury, and decreased inflammatory mediator levels, particularly in renal tissue. Exploratory PK–PD correlations were observed between several compounds and selected pharmacodynamic indicators. Metabolomic analysis suggested disturbances in glycerophospholipid metabolism and the Trp–Kyn pathway in AP rats, some of which were partially reversed after PC treatment. PC showed antibacterial and anti-inflammatory effects in AP rats. Gallic acid, protocatechuic acid, methyl gallate, and quercitrin may be candidate constituents associated with the therapeutic effects of PC, while modulation of glycerophospholipid metabolism and the Trp–Kyn pathway may be involved in its action against AP. These findings provide preclinical pharmacological evidence supporting the therapeutic potential of PC in AP.

## 1. Introduction

Urinary tract infection (UTI) is among the most common bacterial infections worldwide and imposes a substantial burden on patient health and quality of life [1]. Epidemiological studies indicate that approximately 50% of women experience at least one episode of UTI during their lifetime [2]. Most UTIs initially present as cystitis; however, in the absence of timely and effective treatment, pathogens can ascend through the ureters, leading to acute pyelonephritis (AP). In severe cases, the infection may penetrate the renal epithelial and endothelial barriers, resulting in systemic dissemination or sepsis [3]. Therefore, AP represents a critical and potentially preventable stage in the progression of UTI. AP is characterized by an acute inflammatory response involving the renal pelvis and calyces [4], and its clinical manifestations include fever, chills, flank pain, and lower urinary tract symptoms such as frequency, urgency, and dysuria [5,6,7]. While *Escherichia coli* (*E. coli*) and *Klebsiella pneumoniae* are the predominant causative agents, the widespread and often inappropriate use of antibiotics has accelerated the emergence of multidrug resistance. This trend not only compromises therapeutic efficacy but also increases the risk of recurrence, posing a major global health challenge. Because infection is typically accompanied by a pronounced inflammatory response, an ideal therapeutic strategy should combine both antibacterial and anti-inflammatory activities. Consequently, the development of novel agents with dual antibacterial and anti-inflammatory effects has become a major focus in AP and UTI drug discovery.

Traditional Chinese medicine (TCM) and ethnomedicine offer unique advantages in the treatment of UTIs, supported by centuries of clinical practice. Modern pharmacological studies have demonstrated that various TCMs and ethnomedicinal herbs with properties such as releasing the exterior, clearing heat, and eliminating dampness exert therapeutic effects against UTIs through both antibacterial and anti-inflammatory mechanisms [8,9,10,11]. *Polygonum capitatum* Buch.-Ham. ex D. Don (PC), a representative Miao medicinal herb, is a classic natural remedy for UTI [12]. Several PC-based proprietary medicines, including Relinqing granules, capsules, and tablets, have been approved by the China National Medical Products Administration (https://www.nmpa.gov.cn/datasearch/home-index.html#category=yp (accessed on 30 April 2026)) and are widely used in Asia due to their consistent efficacy and favorable safety profile. PC contains diverse bioactive constituent such as flavonoids, phenolic acids, tannins, and volatile oils [13], which exhibit anti-inflammatory, antibacterial, diuretic, and analgesic activities [14,15]. Nevertheless, most studies have emphasized its clinical applications [16], whereas its in vivo pharmacokinetic characteristics—particularly in target tissues—and its precise mechanisms of action remain unclear. In particular, the dual antibacterial and anti-inflammatory effects of PC require further validation. Thus, comprehensive studies integrating chemical characterization, pharmacokinetics, pharmacodynamics, and mechanistic investigation are essential to clarify the pharmacological basis of PC in AP and UTI treatment.

Metabolomics, an essential component of systems biology, employs high-throughput and highly sensitive analytical platforms to comprehensively profile endogenous compounds in biological samples. By applying multivariate statistical analyses, metabolomics can reveal alterations in endogenous metabolic profiles under physiological, pathological, or toxicological conditions [17], thereby facilitating the elucidation of complex metabolic regulatory networks in living systems [18]. Among the various metabolic pathways, tryptophan metabolism, including the indole pathway, serotonin pathway, and kynurenine pathway, plays a particularly important role. Its metabolites are widely involved in inflammatory responses and microbiota regulation, making this pathway a key metabolic route in host–microbe interactions. Studies have shown that this pathway is significantly disrupted in various kidney disease models, such as glomerulonephritis, UTIs, and acute kidney injury, and the levels of its metabolites are closely related to disease severity and recovery progress [19]. Previous studies on PC have mainly focused on its phytochemical composition, pharmacological activities, and the comparative pharmacokinetics of a limited number of constituents in normal and pyelonephritis rats. However, integrated evaluations linking activity-guided extract selection, multi-component pharmacokinetics in both plasma and renal cortex, pharmacodynamic responses, and metabolomic alterations in an AP model remain scarce. Therefore, the present study was designed to provide a broader assessment of the exposure characteristics, therapeutic responses, and pathway-associated metabolic changes of PC in AP. Building upon the optimized extraction process of *Polygonum capitatum* (PC), this study first systematically evaluated the dual anti-inflammatory and antibacterial effects of the extract in an AP rat model. Additionally, the pharmacokinetic profiles of the active components in the renal target tissues and their exposure levels under the pathological state of AP were investigated, along with a pharmacokinetics–pharmacodynamics (PK-PD) correlation analysis of the major active constituents. Finally, metabolomics was employed to explore the changes in renal metabolites before and after treatment with PC in the AP rat model, with the aim of elucidating the metabolic mechanisms underlying its therapeutic effects. This study sought to identify potential biomarkers and metabolic pathways involved in the treatment of AP and UTI with PC. The integrated approach adopted in this work provides valuable experimental evidence for elucidating the pharmacological mechanisms of PC in AP, thereby supporting further development of PC-based medications.

## 2. Results

### 2.1. Optimization of the Extraction Process of PC

**Figure 1:**
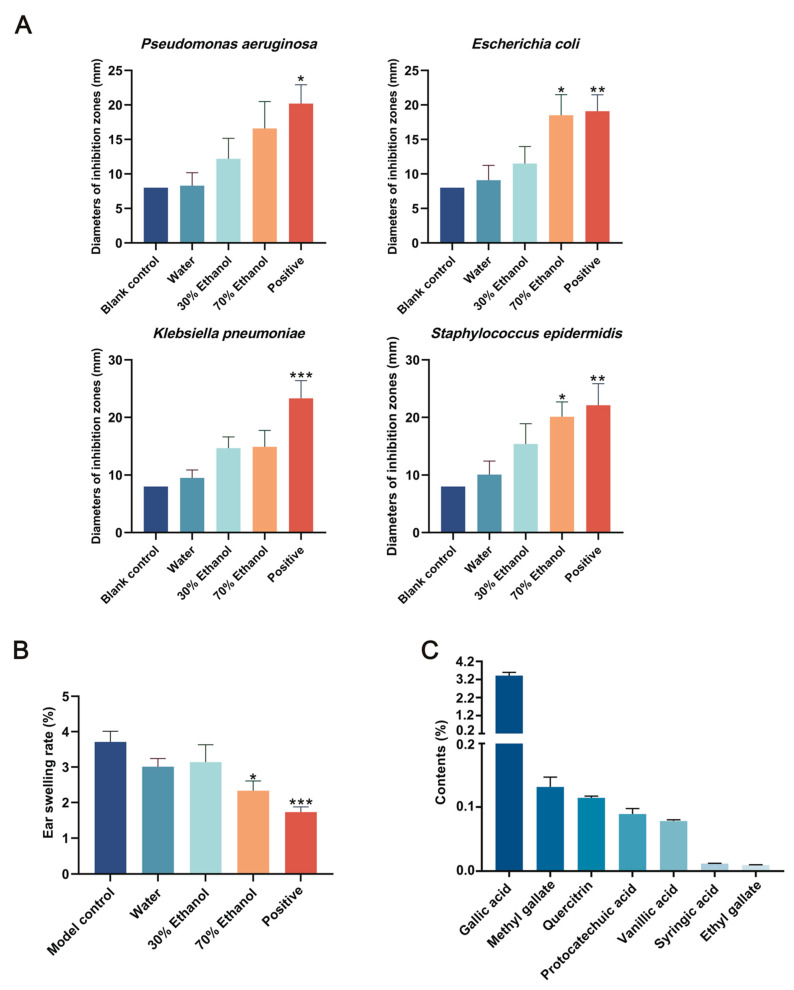
Optimization of *Polygonum capitatum* (PC) extraction process and quantification of extract constituents (**A**). Inhibitory zone diameters of rat medicated serum against different bacterial strains (**B**). Effects of different PC extracts on xylene-induced ear swelling in mice. (**C**). The contents of seven major constituents in the 70% ethanol extract of PC. * *p* < 0.05, ** *p* < 0.01, *** *p* < 0.001 (compared to the blank control group).

In the in vitro antibacterial assay, compared with the blank control group, both the positive control and the PC extracts produced larger inhibition zones against the four tested bacterial strains. Among the drug-containing serum groups, the 70% ethanol extract of PC exhibited the strongest antibacterial activity against all tested strains and showed statistically significant differences (*p* < 0.05) against *E. coli* and *Staphylococcus epidermidis* compared with the blank control group. This activity was superior to that of the water extract and the 30% ethanol extract, indicating that the 70% ethanol extract showed the highest antibacterial activity in the present screening assay. The inhibition zone diameters are shown in Figure 1A.

In the xylene-induced mouse ear edema model, all PC extracts significantly reduced ear swelling compared with the blank control group. Among the treatment groups, the 70% ethanol extract of PC demonstrated the greatest inhibitory effect (*p* < 0.05), outperforming both the water and 30% ethanol extracts, suggesting that it possessed the strongest anti-inflammatory activity. The ear swelling results are presented in Figure 1B.

Therefore, the 70% ethanol extract was selected for subsequent constituent analysis and further in vivo investigation. The extraction yield of the optimized 70% ethanol extraction procedure was 7%, which was subsequently used as a reference for estimating the equivalent extract dose in the animal experiments.

### 2.2. Quantitative Determination of Multiple Constituents in the PC Extract

The validation results of the analytical method for content determination demonstrated that the precision, repeatability, stability, and recovery of all tested compounds met the requirements of the Guidelines for Bioanalytical Method Validation [20]. Detailed validation results are provided in the Supplementary Materials (Quantitative methodology validation results). The quantitative results of the seven constituents in the 70% ethanol extract of PC are shown in Figure 1C. The relative contents of the seven compounds in the extract were ranked as follows: gallic acid (3.4168%) > methyl gallate (0.1315%) > quercitrin (0.1145%) > protocatechuic acid (0.0892%) > vanillic acid (0.0782%) > syringic acid (0.0118%) > ethyl gallate (0.0095%). The total content of the seven major constituents was 3.8515%. Among these, gallic acid exhibited the highest content, accounting for 3.4168% of the extract.

### 2.3. Pharmacokinetic Results

#### 2.3.1. Method Validation Results

None of the seven analytes or the internal standard showed interference from endogenous substances, demonstrating good specificity of the method, as shown in the representative chromatograms in Figure S1A–G.

The calibration curves, linear ranges, accuracy, precision, and stability of the analytes in biological samples were validated according to the Guidelines for Bioanalytical Method Validation [20]. The results met the methodological requirements and were sufficient for quantifying the compounds in plasma and renal microdialysates after oral administration of the PC extract. Detailed validation data are provided in the Supplementary Materials (Pharmacokinetic methodology validation results; Table S2).

#### 2.3.2. Pharmacokinetics of PC in Plasma and Renal Microdialysates

**Figure 2:**
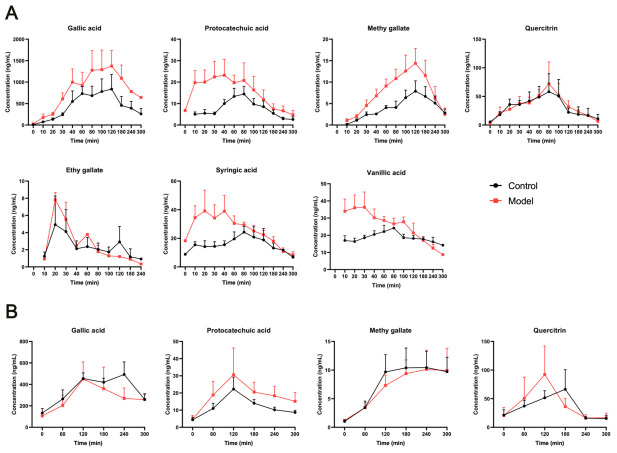
Plasma and renal cortex concentration–time profiles of major compounds after *Polygonum capitatum* (PC) treatment in normal and AP rats. (**A**) Plasma; (**B**) Renal cortex.

The plasma concentration–time profiles of the analytes in normal and AP rats are shown in Figure 2A, and the pharmacokinetic parameters are summarized in Table 1. Notably, gallic acid, protocatechuic acid, syringic acid, and quercitrin remained detectable in plasma prior to dosing on day 7. Complete pharmacokinetic curves were obtained for all seven analytes, except ethyl gallate, which exhibited low plasma concentrations and insufficient data for reliable parameter calculation. In normal rats, the time to peak plasma concentration (T_max_) ranged from 45.0 ± 32.7 min for syringic acid to 116.7 ± 58.5 min for methyl gallate. The area under the plasma concentration–time curve (AUC_last_) ranked as follows: gallic acid > quercitrin > vanillic acid > syringic acid > protocatechuic acid > methyl gallate, with gallic acid showing the highest exposure (136,855.3 ± 111,662.4 ng·mL^−1^·min). Both C_max_ and AUC_last_ followed the same trend. Compared with normal rats, AP rats showed comparable T_max_ values but significantly elevated C_max_ and AUC for most analytes (*p* < 0.05). The apparent volume of distribution (*Vz*/*F*) was markedly reduced in AP rats, suggesting altered tissue distribution may underlie the increased plasma exposure.

The renal cortical concentration–time curves are presented in Figure 2B, and pharmacokinetic parameters are listed in Table 2. Among the detected compounds, gallic acid, quercitrin, protocatechuic acid, and methyl gallate exhibited complete pharmacokinetic curves in renal tissue, while vanillic acid and syringic acid did not. For all compounds, T_max_ in the renal cortex was delayed relative to that in plasma, indicating slower tissue distribution. Gallic acid showed the highest renal exposure, with C_max_ and AUC_last_ of 521.5 ± 153.6 ng·mL^−1^ and 104,955.1 ± 24,776.9 ng·mL^−1^·min, respectively, in normal rats.

In AP rats, T_max_ was significantly prolonged for quercitrin (111.4 ± 41.4 vs. 210.0 ± 103.9 min, *p* < 0.05), while other compounds showed no significant change. Renal C_max_ values decreased for gallic acid and methyl gallate but increased for protocatechuic acid and quercitrin, with AUC values consistent with these trends. These results indicate that the pathological state of AP substantially alters the pharmacokinetic behavior of PC constituents in both plasma and renal tissue.

### 2.4. Pharmacodynamic Results

#### 2.4.1. Effects of PC on Urinary Bacterial Culture in AP Rats

Bacterial culture of urine samples revealed a 0% positive rate in the sham group and 100% in the model group. This significant increase (*p* < 0.01) confirmed the successful establishment of bacterial infection and the validity of the AP model. In contrast, PC treatment reduced the positive rate to 37.5%, which was significantly lower than that in the model group (*p* < 0.05). These findings demonstrate that PC exerts a marked antibacterial effect, likely by alleviating UTI and inhibiting pathogen proliferation. Detailed data on positive cases and rates are presented in Table S3.

#### 2.4.2. Effects of PC on Routine Urinary Parameters in AP Rats

In the model group, the incidence of PRO-positive was significantly higher than that in the sham group on days 1, 3, and 7 (*p* < 0.001), indicating renal injury and impaired glomerular filtration. Specifically, PRO-positive rates in the model group were 75% on day 3 and 62.5% on day 7, while both were reduced to 37.5% in the PC-treated group. Compared with the model group, PC treatment significantly lowered the proportion of PRO-positive animals on both days 3 and 7 (*p* < 0.05), suggesting a protective effect against proteinuria. Detailed results are provided in Table S4.

Other urinary indicators—including BLD, KET, GLU, BIL, and URO—showed no significant abnormalities before or after modeling and treatment.

#### 2.4.3. Effects of PC on Renal Function Indicators in AP Rats

**Figure 3:**
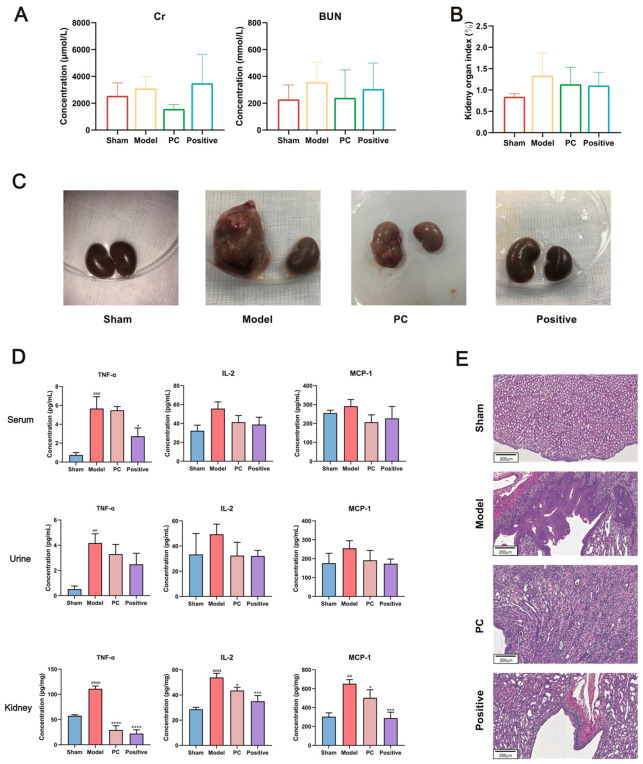
Evaluation of the therapeutic efficacy of *Polygonum capitatum* (PC) in AP rats. (**A**) Effects of PC on serum blood urea nitrogen (BUN) and creatinine (Cr). (**B**) Effects of PC on renal organ index. (**C**) Gross morphology of kidneys in different groups. (**D**) Levels of inflammatory mediators in serum, urine, and renal tissue. (**E**) Histopathological analysis of renal tissue by H&E staining. ^##^ *p* < 0.01, ^###^ *p* < 0.001 and ^####^ *p* < 0.0001 (compared to the sham group). * *p* < 0.05, *** *p* < 0.001 and **** *p* < 0.0001 (compared to the model group).

Serum biochemical parameters revealed marked elevations of blood urea nitrogen (BUN) and serum creatinine (Scr) in the model group compared with the sham group, reflecting impaired renal parenchymal and excretory function. Treatment with PC significantly reduced both BUN and Scr levels relative to those in the model group (Figure 3A), indicating a protective effect against AP-induced renal dysfunction.

#### 2.4.4. Effects of PC on Renal Morphology and Organ Index in AP Rats

The renal organ index was significantly increased in model rats, consistent with renal swelling and inflammation. PC administration reduced both the bilateral kidney weight ratio and the renal organ index compared with the model group (Figure 3B; Table S5).

Morphological assessment further supported these results. Kidneys from sham-operated rats displayed a normal appearance, smooth surfaces, and clear corticomedullary boundaries. In contrast, model rats exhibited marked pathological changes, including enlargement of the left kidney (infected side), irregular cortical scarring, mucosal congestion, and the presence of purulent exudate in the pelvis. In PC-treated rats, only mild abscess formation was observed, accompanied by reduced congestion and swelling, while kidneys from the positive control group appeared nearly normal, with only slight enlargement compared to the contralateral side (Figure 3C).

#### 2.4.5. Effects of PC on Immune Factors in Serum, Urine, and Renal Tissue

Changes in inflammatory cytokines are illustrated in Figure 3D. Compared with the sham group, serum TNF-α levels were markedly increased in the model group, while IL-2 and MCP-1 also displayed upward trends, indicating the presence of systemic inflammatory responses in AP rats. After PC treatment, the levels of TNF-α, IL-2, and MCP-1 in serum were reduced to varying degrees; however, not all of these changes reached statistical significance.

In urine samples, TNF-α levels were significantly elevated in the model group, while IL-2 and MCP-1 showed similar upward trends. After PC treatment, urinary TNF-α was reduced, and IL-2 and MCP-1 also showed trends toward reduction; however, the statistical support varied among the three indicators.

In renal tissues, TNF-α, IL-2, and MCP-1 concentrations were significantly elevated in model rats compared with the sham group. PC treatment markedly downregulated these inflammatory mediators, demonstrating its strong anti-inflammatory effect and ability to suppress renal inflammatory responses.

#### 2.4.6. Effects of PC on Renal Histopathology in AP Rats

Histological examination using hematoxylin–eosin staining (Figure 3E) revealed normal renal architecture in sham-operated rats, characterized by intact glomeruli, tubular structures, and the absence of inflammatory infiltration. In contrast, the model group displayed severe pathological lesions, including renal pelvic dilation, epithelial hyperplasia with necrosis and suppuration, tubular dilation, extensive interstitial inflammatory cell infiltration, and multiple microabscesses, confirming successful model establishment.

PC treatment markedly ameliorated these pathological injuries, as evidenced by reduced tubular dilation, alleviated interstitial inflammation, and fewer infiltrating inflammatory cells. These results demonstrate that PC confers notable protection against renal inflammation and tissue injury induced by bacterial infection.

### 2.5. PK–PD Correlation Analysis

Exploratory integration of the pharmacokinetic and pharmacodynamic data revealed compound-specific relationships between systemic exposure and biological responses (Table 3). Among the seven analytes, six—gallic acid, protocatechuic acid, vanillic acid, quercitrin, ethyl gallate, and methyl gallate—displayed notable correlations with key PD markers.

Protocatechuic acid exhibited the highest correlation with urinary protein (PRO; r = 0.664), whereas vanillic acid showed the highest correlation with ketone bodies (KET; r = 0.632). For TNF-α, correlation coefficients greater than 0.5 were observed for gallic acid, protocatechuic acid, and vanillic acid. IL-2 showed correlation coefficients greater than 0.5 with protocatechuic acid, quercitrin, ethyl gallate, and methyl gallate, whereas MCP-1 showed relatively strong correlations with gallic acid, protocatechuic acid, and quercitrin.

Collectively, these exploratory results suggest that gallic acid exhibited broader exposure–response associations across multiple PD indicators than the other measured compounds. However, these correlations should be interpreted cautiously and do not by themselves establish causality for the antibacterial or anti-inflammatory effects of PC extract in AP.

### 2.6. Metabolomics Results

#### 2.6.1. Establishment of Renal Metabolomic Profiles

Renal tissue metabolites were profiled using an HPLC-Orbitrap/MS system, and representative chromatograms were acquired in both positive and negative ionization modes (Figure S2). The high degree of overlap in retention times and peak areas among samples indicated good chromatographic reproducibility and analytical stability. These results support the quality and reliability of the metabolomics dataset for subsequent statistical analyses.

#### 2.6.2. Screening of Differential Metabolites

**Figure 4:**
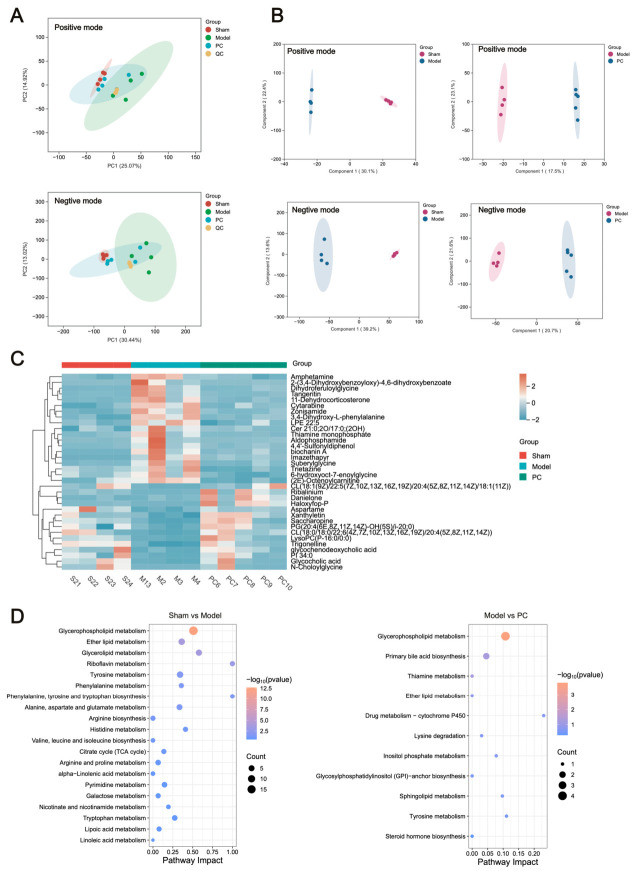
Metabolite changes in AP rats following the *Polygonum capitatum* (PC) intervention. (**A**) PCA plots in kidney samples. (**B**) OPLS-DA scores scatter plot of kidney samples. (**C**) Distribution of the kidney tissue differential metabolites in each group. (**D**) The metabolic pathways of differential metabolites in kidney samples.

Unsupervised PCA was initially performed to evaluate data distribution and system performance (Figure 4A). The tight clustering of QC samples indicated excellent analytical stability and reproducibility. A clear separation was observed between the model group and both the sham and PC-treated groups, reflecting pronounced metabolic perturbations induced by AP and substantial metabolic recovery following PC intervention.

To further identify potential biomarkers, OPLS-DA was conducted between the sham and model groups, as well as between the model and PC groups, in both positive and negative ion modes (Figure 4B). The distinct clustering in OPLS-DA score plots confirmed marked differences in metabolic profiles across groups.

Using the selection criteria of VIP > 1, FC > 1.5 or < 0.7, and *p* < 0.05, a total of 743 differential metabolites were identified between the sham and model groups, including 369 upregulated and 374 downregulated metabolites. Comparison between the model and PC groups revealed 34 differential metabolites, comprising 19 upregulated and 15 downregulated metabolites (Figure 4C). These findings indicate that PC treatment was associated with partial modulation of the metabolic disturbances induced by AP.

#### 2.6.3. Metabolic Pathway Analysis

To explore metabolic pathways associated with AP and the response to PC treatment, pathway enrichment and topology analyses were performed using the MetaboAnalyst 6.0 platform. Pathways with an impact value greater than 0 were regarded as biologically meaningful.

In renal tissue samples, 55 metabolic pathways were altered in the model group, primarily involving glycerophospholipid metabolism, ether lipid metabolism, glycerolipid metabolism, riboflavin metabolism, and tyrosine metabolism. These disruptions are closely associated with renal inflammation, oxidative stress, and membrane damage in AP.

**Figure 5:**
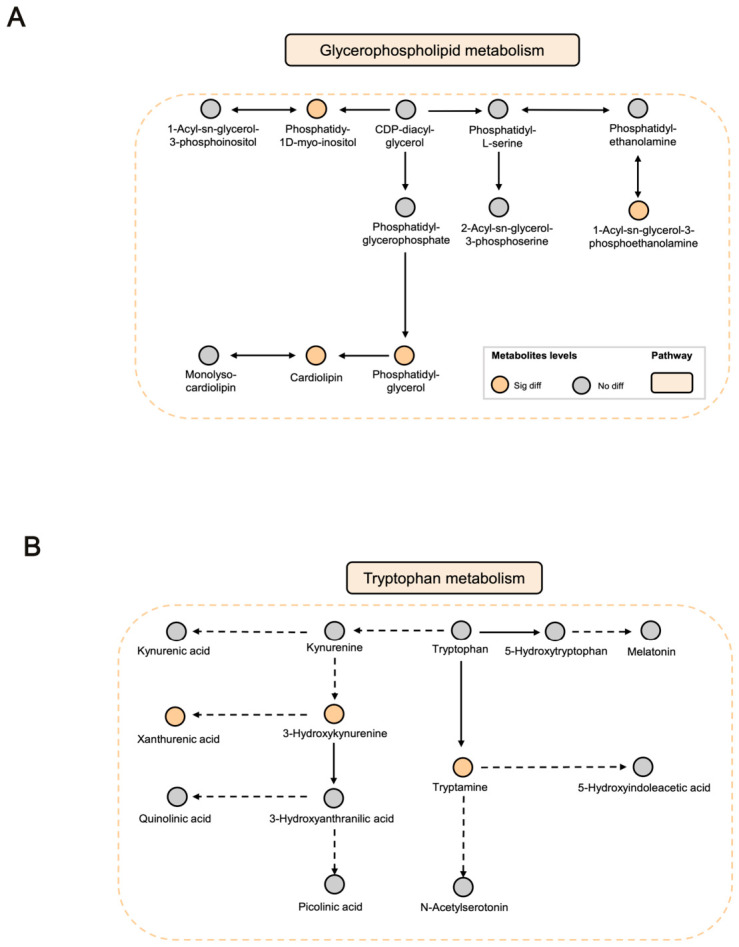
*Polygonum capitatum* (PC) intervened in the key metabolic pathways of the AP rats. (**A**) Glycerophospholipid metabolism. (**B**) Tryptophan metabolism.

After PC treatment, 11 metabolic pathways were modulated, among which glycerophospholipid metabolism, primary bile acid biosynthesis, ether lipid metabolism, and tyrosine metabolism showed relatively prominent changes (Figure 4D). Among these, glycerophospholipid metabolism was one of the major pathways highlighted by the untargeted metabolomics analysis, with representative metabolites mapped in Figure 5A.

Furthermore, targeted metabolomics focusing on tryptophan metabolism revealed significant alterations in the kynurenine branch, indicating that PC may also restore immune–metabolic homeostasis by regulating the tryptophan–kynurenine axis (Figure 5B).

#### 2.6.4. Alterations in Key Metabolites

**Figure 6:**
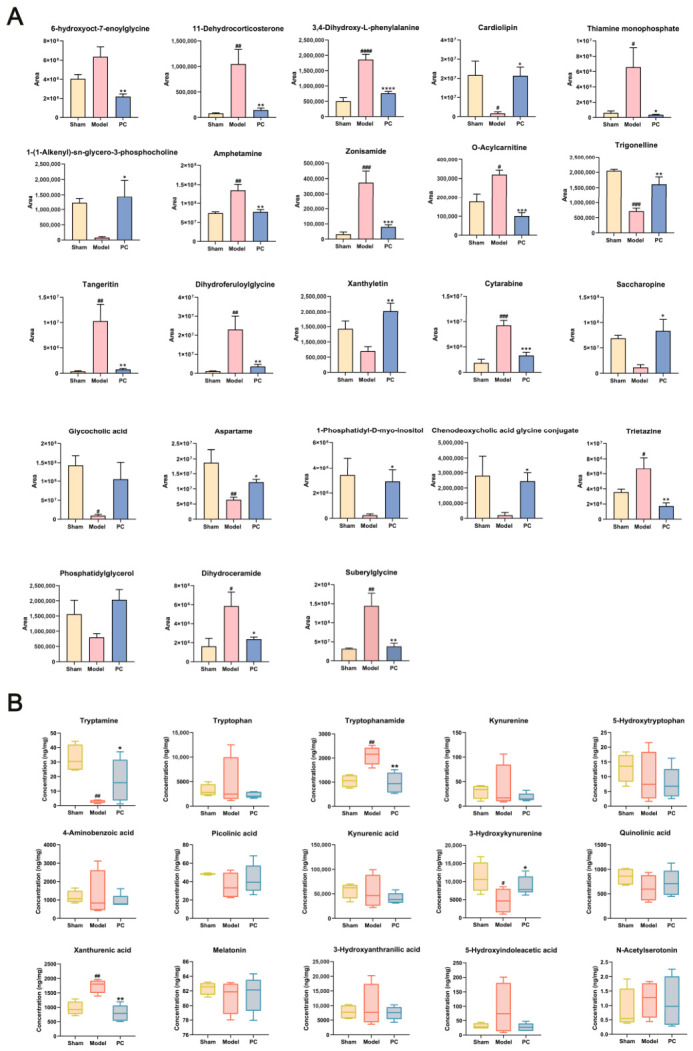
*Polygonum capitatum* (PC) altered the metabolites of the kidney samples in the AP rats. (**A**). Results of key metabolite levels determined by untargeted metabolomics (**B**). Results of key metabolite levels determined by tryptophan–kynurenine-targeted metabolomics ^#^ *p* < 0.05, ^##^ *p* < 0.01, ^###^ *p* < 0.001 and ^####^ *p* < 0.0001 (compared to the sham group). * *p* < 0.05, ** *p* < 0.01, *** *p* < 0.001 and **** *p* < 0.0001 (compared to the model group).

In the untargeted metabolomics analysis of renal tissue, the model group exhibited significantly increased levels of metabolites such as 3,4-dihydroxy-L-phenylalanine, thiamine monophosphate, O-acylcarnitine, and amphetamine, while levels of cardiolipin, trigonelline, aspartame, and glycocholic acid were markedly reduced. Following PC treatment, these alterations were partially reversed to varying degrees (Figure 6A).

Targeted metabolomics focusing on the tryptophan metabolic pathway revealed elevated concentrations of Trp, Trn, Kyn, 4-AA, KA, and XA in the model group, accompanied by reduced levels of TrpA and 3-HK. PC administration resulted in partial normalization of these alterations, indicating that the treatment mitigated the dysregulation of tryptophan–kynurenine metabolism associated with AP (Figure 6B).

## 3. Discussion

TCM offers unique advantages in treating infectious diseases such as AP, owing to its multi-component and multi-target synergistic mechanisms. Previous studies have shown that aqueous extracts of PC exhibited notable antibacterial, anti-inflammatory, and antioxidant activities. Similarly, ethyl acetate and 95% ethanol extracts have also demonstrated strong antioxidant, anti-inflammatory, and antibacterial activities [16]. The 70% ethanol extract of PC, as well as its major constituents—including gallic acid and its derivatives, flavonoids, tannins, triterpenes, and steroids—has also been shown to significantly reduce inflammation and bacterial growth [21,22]. Moreover, PC extract exerts dose-dependent anti-inflammatory efficacy within 3–6 g/kg. Administration at 6 g/kg for 7 days produced no observable toxicity such as convulsion, ataxia, diarrhea, or diuresis [21]. Although ethanol is a widely utilized and safe solvent in phytochemical extraction, the efficacy variations among different ethanol-based extracts of PC have not yet been fully elucidated. In this study, we prepared aqueous, 30% ethanol, and 70% ethanol extracts of PC and found the 70% ethanol extract (0.42 g/kg, equivalent to 6 g crude herb/kg) to be the most effective. It showed superior antibacterial performance against all four tested strains and markedly inhibited xylene-induced ear edema in mice. These results align with existing reports, confirming the potent anti-inflammatory and antibacterial properties of the 70% ethanol extract [12,21]. It should be noted that ethanol itself has antibacterial activity. Because no independent solvent control was included in the present extract-screening experiment, the contribution of residual ethanol to the observed antibacterial differences among extracts cannot be fully excluded.

Compared with previous studies that mainly focused on the comparative pharmacokinetics of a limited number of constituents, the present work extended the analysis to seven major compounds and further integrated pharmacodynamic evaluation, exploratory PK–PD correlation analysis, and both untargeted and targeted metabolomics in the AP model. Furthermore, our previous phytochemical studies isolated 30 monomeric compounds from PC. In this study, seven compounds with relatively high contents and previously reported pharmacological activities—gallic acid, protocatechuic acid, vanillic acid, syringic acid, ethyl gallate, methyl gallate, and quercitrin—were selected for quantitative analysis. All seven compounds were detected in the 70% ethanol extract of PC. Pharmacokinetic analysis demonstrated that these compounds were absorbed into the systemic circulation after oral administration, while gallic acid, protocatechuic acid, methyl gallate, and quercitrin were also detected in the renal cortex, indicating renal exposure of these constituents. Notably, the pathological state of AP altered the pharmacokinetic profiles of several measured constituents, which may be associated with AP-related changes in renal function and systemic disposition. Exploratory PK–PD correlation analysis further showed that six compounds—gallic acid, protocatechuic acid, vanillic acid, quercitrin, ethyl gallate, and methyl gallate—had relatively strong correlations (correlation coefficients > 0.5) with several pharmacodynamic indicators, including PRO, KET, TNF-α, IL-2, and MCP-1. Taken together, these findings suggest that gallic acid, protocatechuic acid, methyl gallate, and quercitrin may represent candidate constituents associated with the therapeutic effects of PC. However, their direct contributions to the anti-inflammatory and antibacterial effects of PC require further validation.

In this study, a retrograde infectious AP model was established in rats by intravesical inoculation with pathogenic *E. coli*. This model closely mimics the pathological progression of AP in humans caused by UTIs. Positive urine culture is considered the gold standard for AP diagnosis. In addition, routine urine indicators, such as the presence of PRO and KET, also reflect renal injury. The renal index, calculated from kidney weight, is an indicator of renal impairment and typically increases during bacterial infection due to inflammatory cell infiltration, fibroblast proliferation, and edema, correlating positively with kidney swelling and tissue damage. Elevated serum Cr and BUN levels—end products of nitrogen metabolism cleared via glomerular filtration—indicate impaired renal function, as do positive urinary protein and ketone results. In our experiment, AP model rats showed significantly higher rates of positive urine cultures, elevated urinary protein levels, increased renal index, and higher serum BUN and Cr levels, confirming successful model establishment. After PC intervention, the positive urine culture rate, urinary PRO, and serum BUN and Cr levels were all reduced, demonstrating its antibacterial and nephroprotective effects. However, PC showed limited improvement in the renal index, possibly due to the short treatment duration, incomplete recovery of the renal parenchyma, or compensatory renal hypertrophy during pathological states.

Inflammatory responses are an important pathological feature of AP and are characterized by elevated levels of pro-inflammatory cytokines both systemically and locally. TNF-α, as a pivotal pro-inflammatory mediator, can activate neutrophils and orchestrate the inflammatory cascade [23,24]. IL-2 is primarily produced by activated T cells and promotes the proliferation and differentiation of lymphocytes [25,26]. MCP-1, secreted by monocytes and macrophages, contributes to the recruitment and activation of inflammatory cells [27,28]. In the present study, serum levels of these inflammatory cytokines were elevated in AP model rats, indicating a successful induction of systemic inflammatory responses. PC treatment reduced the levels of these cytokines. Notably, the statistical support for these changes was stronger in renal tissue than in serum, suggesting that the anti-inflammatory effects of PC were more evident at the tissue level under the present experimental conditions. Therefore, the inflammatory indicators in this study should be interpreted as supportive evidence of pharmacological improvement, rather than as stand-alone evidence for a definitive mechanism. Together with the improvements in urinary bacterial load, renal function, and histopathological injury, these findings support the overall therapeutic potential of PC in AP.

Dysregulated host metabolism induced by *E. coli* infection may contribute to the onset and progression of AP, while inflammatory response may further aggravate these metabolic disturbances. Host–microbe interactions mediated through metabolites form a complex, multi-step and multi-layered pathological network underlying AP. In the present study, metabolomics was used to characterize endogenous metabolic alterations associated with AP and the response to PC treatment, thereby providing clues to the pathological processes of AP and the metabolic basis of drug intervention. Untargeted pathway enrichment analysis indicated that glycerophospholipid metabolism plays a pivotal role in both the development of AP and its modulation by PC treatment. Lipid metabolic disorders are closely associated with the progression of renal diseases; abnormal lipid accumulation disrupts cellular homeostasis and activates lipid synthesis and inflammation-related pathways, leading to tubular injury, apoptosis, and tissue fibrosis, which collectively accelerate renal dysfunction [29]. Glycerophospholipids are major components of cellular membranes and are involved in membrane signaling and metabolic regulation [30,31], and their dysregulation is strongly linked to inflammation [32]. Cardiolipin (CL), a key glycerophospholipid located in the inner mitochondrial membrane, is essential for maintaining the structural integrity and functional stability of oxidative phosphorylation complexes. Disturbed CL metabolism can induce mitochondrial dysfunction, lipid peroxidation, and lipid droplet accumulation, thereby contributing to renal injury. In this study, several metabolites related to glycerophospholipid metabolism were altered in AP rats, including decreased 1-acyl-sn-glycero-3-phosphoethanolamine and elevated phosphatidylglycerol (PG), phosphatidy-1d-myo-inositol (PI), and CL. These findings suggest that glycerophospholipid metabolism plays a critical role in the pathogenesis of AP. Importantly, PC treatment partially reversed some of these changes, suggesting that modulation of glycerophospholipid metabolism may be involved in its therapeutic effects.

In addition to glycerophospholipid metabolism, tryptophan metabolism also plays a crucial role in kidney diseases and host–microbe interactions. Our targeted metabolomics analysis demonstrated that PC treatment partially restored the abnormal levels of several metabolites within the Trp–Kyn pathway, suggesting that the Trp–Kyn axis may be involved in the metabolic response to PC treatment. Increasing evidence indicates that *E. coli* infection can disrupt the Trp–Kyn pathway and thereby influence inflammatory progression. For example, kynurenine has been reported to promote inflammation [33], whereas indole-3-lactic acid may exert anti-inflammatory effects [34]. Indoleamine 2,3-dioxygenase (IDO), the rate-limiting enzyme of the Trp–Kyn pathway, is induced during UTI caused by *E. coli* and suppresses neutrophil chemotaxis through kynurenine production, thereby dampening host innate immunity. Moreover, IDO expression is markedly upregulated in several kidney injury models, including acute kidney injury and crescentic glomerulonephritis, while its inhibition aggravates renal damage, suggesting a potential protective role of this pathway [35]. Clinical studies have also shown that urinary levels of tryptophan and its metabolites are closely associated with the recovery process in patients with acute kidney injury [36]. Taken together, these findings suggest that alterations in the Trp–Kyn pathway may be associated with the response to PC treatment in AP, although the precise mechanisms underlying this regulation warrant further investigation.

This study has several limitations. The in vivo AP model was established using only *E. coli*, which, although being one of the most common causative pathogens of UTI, does not represent the full spectrum of bacteria involved in pyelonephritis. In addition, the study was performed in an acute AP model with a 7-day treatment period, so the findings may not fully reflect the effects of PC under chronic or recurrent infectious conditions. Only a single dose of PC extract (0.42 g/kg) was evaluated, and no formal dose–response analysis was carried out. Moreover, no independent toxicity or safety assessment was included. Although PC-related preparations have been used clinically, this background cannot substitute for a dedicated safety evaluation of the specific 70% ethanol extract and dosing regimen used here. In addition, the PK–PD correlation analysis should be interpreted with caution, as it was based on a limited sample size and no formal multiple-comparison correction was applied. Taken together, these considerations indicate that the present findings should be interpreted within the scope of a preclinical exploratory study. Future work should address these limitations by including additional clinically relevant pathogens, longer-term or chronic infection models, systematic dose–response studies, dedicated toxicity and safety assessments, functional validation of pathway-associated targets, and improved analytical methods for low-abundance constituents.

## 4. Materials and Methods

### 4.1. Animals

All experimental procedures were approved by the Animal Ethics Committee of the Medical Experimental Center, China Academy of Chinese Medical Sciences (Approval No.: ERCCACMS21-2111-26). Animals were housed in a specific pathogen-free (SPF) facility under controlled conditions (temperature: 20–25 °C; relative humidity: 45–65%; light/dark cycle: 12 h/12 h) with free access to food and water. Three animals were housed per cage, and all were acclimatized for at least 3 days before the experiments.

Male Kunming mice (4–6 weeks old, 20–25 g) were purchased from SPF (Beijing) Biotechnology Co., Ltd. (Beijing, China). Male Sprague Dawley (SD) rats (8–9 weeks old, 250–300 g) were obtained from Beijing Vital River Laboratory Animal Technology Co., Ltd. (Beijing, China).

### 4.2. Reagents and Materials

*Polygonum capitatum* Buch.-Ham. ex D. Don (whole herb, Polygonaceae) was purchased from Guizhou Warmen Pharmaceutical Co., Ltd. (Guiyang, China) and authenticated as genuine by Chief Pharmacist Xirong He, Institute of Chinese Materia Medica, China Academy of Chinese Medical Sciences.

Indomethacin capsules (Batch No.: 20180120) were purchased from Hebei Yongfeng Pharmaceutical Co., Ltd. (Shijiazhuang, China); levofloxacin tablets (Batch No.: BA190G1) from Daiichi Sankyo Pharmaceutical (Beijing) Co., Ltd. (Beijing, China); and penicillin sodium for injection (Batch No.: 20171217) from Jingxin Pharmaceutical Co., Ltd. (Weifang, China). Isoflurane (lot number: 217180801) was purchased from RWD Life Science Co., Ltd. (Guangdong, China). Sodium pentobarbital (lot number: 850601) was purchased from Guizhou Merck Chemical Reagent Co., Ltd. Chemical Reagent Branch (Tongren, China).

Reference bacterial strains—including *E. coli* ATCC 25922, *Staphylococcus aureus* ATCC 25923, *Streptococcus pneumoniae* BNCC 337114, and *Pseudomonas aeruginosa* ATCC 27853—were all obtained from BeNa Culture Collection (Beijing, China).

Standards of gallic acid, protocatechuic acid, vanillic acid, syringic acid, methyl gallate, ethyl gallate, and quercitrin were isolated and identified by our laboratory. Bergeninum (internal standard) was purchased from J&K Scientific Ltd. (Beijing, China). The purity of all compounds was more than 98% (as determined by HPLC).

Instruments and equipment included: Microdialysis probes (MAB 7.8.10, MAB, Sweden); UV–Vis spectrophotometer (T95, Persee General Instrument Co., Beijing, China); Urine analyzer (URIT-150Vet, URIT Electronic Co., Guilin, China); Multi-Mode microplate readers (Synergy H1 and Synergy2, BioTek, Winooski, VT, USA); Automatic tissue processor (Tissue-Tek VIP 5 Jr, Sakura, Japan); embedding machine (1150H, Leica, Wetzlar, Germany); Rotary microtome (RM2235, Leica, Wetzlar, Germany); tissue flotation bath (Hi1210, Leica, Wetzlar, Germany); Automated stainer and coverslipper (Prisma E2s and Film E2, Sakura, Japan); Whole-slide scanner (Digital Slide Scanner ProScanner APro 5, AMOS Scientific Pty. Ltd., Melbourne, Australia); Multi-tube vortexer mixer (Targin VX-III, Beijing Targin Technology Co., Beijing, China); –86 °C ultra-low-temperature freezer (Forma 88000 series, Thermo Scientific, Waltham, MA, USA); High-speed refrigerated centrifuge (Rotanta 460R, Hettich, Germany); Vacuum centrifugal concentrator (MC-8, Beijing GM Technology Co., Beijing, China); Desktop anesthesia machine (Harvard Apparatus, Holliston, MA, USA); Body temperature maintenance system (ThermoStar, RWD Life Science Co., Shenzhen, China); and Independent ventilated cages (EasyFlow, Tecniplast, Buguggiate, Italy).

Analytical systems included: HPLC-UV system for quantitative analysis, consisting of an LC-20A system (Shimadzu, Kyoto, Japan) equipped with an SPD-20A photodiode array detector. LC–MS/MS system for pharmacokinetic analysis: 1290 Infinity II HPLC coupled to a Triple Quad 6460 tandem mass spectrometer (Agilent, Santa Clara, CA, USA) with MassHunter Quantitative Analysis software version 12.1. HPLC–Orbitrap/MS system (Thermo Fisher Scientific, Waltham, Massachusetts, USA) for untargeted metabolomic study, consisting of a U3000 UHPLC, Q Exactive™ hybrid quadrupole-Orbitrap mass spectrometer, and Xcalibur 3.0 software for data acquisition and processing. LC-MS/MS system (AB Sciex, Marlborough, MA, USA) for targeted metabolomic study, consisting of an ExionLC-20AC HPLC, IonDrive™ Turbo V ion source, Sciex 6500+ triple quadrupole mass spectrometer, Analyst 1.7 software for data acquisition, and MultiQuant 3.0.3 for data processing.

ELISA kits for TNF-α, IL-2, and MCP-1 were purchased from CUSABIO^®^ (Wuhan Huamei Biotechnology Engineering Co., Ltd., Wuhan, China).

Metabolite standards and reagents used for targeted metabolomics included pathway-related reference compounds, sodium tetraborate, benzoyl chloride, and d5-benzoyl chloride (all purchased from Sigma, St. Louis, MI, USA). All reference and internal standards were of >99% purity. LC/MS-grade acetonitrile, methanol, formic acid, and ammonium formate were obtained from Beijing Dikma Technologies Inc. (Beijing, China).

### 4.3. Optimization of the PC Extraction Process

The extraction procedure of PC was preliminarily optimized based on antibacterial activity of drug-containing serum in vitro and anti-inflammatory activity in the mouse ear swelling assay. PC raw material (10 kg, coarse powder) was extracted with 8 volumes of solvent (water, 30% ethanol, or 70% ethanol), soaked for 1 h, and then refluxed under reduced pressure for 1.5 h. The residue was subsequently re-extracted with 6 volumes of the same solvent under reduced pressure for another 1.5 h. The two extracts were combined, concentrated under vacuum to a defined volume, and lyophilized to yield the final extract (~0.7 kg).

#### 4.3.1. Evaluation of Antibacterial Activity

SD rats were randomly divided into five groups (*n* = 5): blank control, PC water extract (0.42 g/kg), PC 30% ethanol extract (0.42 g/kg), PC 70% ethanol extract (0.42 g/kg), and positive control (penicillin, 10 mg/kg). With the exception of the blank control group, which received purified water, all groups were administered the corresponding extract twice daily by oral gavage for two consecutive days. The positive control group received intraperitoneal injections of penicillin once on the day before and once on the day of the experiment.

One hour after the final administration, blood was collected from the abdominal aorta to prepare drug-containing serum under general anesthesia (induced with 5% isoflurane and maintained with 2.5% isoflurane). Pathogenic strains including *Staphylococcus aureus*, *Klebsiella pneumoniae*, *Pseudomonas aeruginosa*, and *E. coli* were cultured on nutrient agar plates (20 mL per plate). Sterile stainless-steel perforators (6 mm in diameter) were used to punch two equidistant wells in the agar. Each well was filled with one drop of molten agar containing 100 μL of drug-containing serum. Normal saline served as the blank control. The plates were incubated at 37 °C for 24 h, and the diameters of the inhibition zones were measured [37]. The antibacterial activities of the different PC extracts were evaluated on the basis of inhibition zone diameter, with four replicates per group.

#### 4.3.2. Anti-Inflammatory Activity Evaluation in the Mouse Ear Swelling Test

Kunming mice were randomly divided into five groups (*n* = 10): model control, PC water extract (0.42 g/kg), PC 30% ethanol extract (0.42 g/kg), PC 70% ethanol extract (0.42 g/kg), and positive control (indomethacin, 10 mg/kg). Mice in the extract groups received the corresponding extract by daily oral gavage for three consecutive days, while the blank control group received an equal volume of purified water. The positive control group was administered indomethacin once on the day of modeling.

One hour after the final administration, 25 μL of xylene was applied to both surfaces of the right ear to induce swelling. After 90 min, mice were sacrificed by cervical dislocation, and 8 mm ear punches were taken from identical positions of both ears. The ear swelling rate was calculated as follows: Ear swelling rate (%) = (Weight of right ear punch –Weight of left ear punch)/Weight of left ear punch ×100% [38]. The anti-inflammatory activities of the different PC extracts were evaluated on the basis of the ear swelling rate.

### 4.4. Quantification of Chemical Constituents in the 70% Ethanol Extract of PC

Based on the preliminary activity-guided screening results, the 70% ethanol extract was selected as the final study extract for subsequent chemical characterization and in vivo investigations. The contents of seven major compounds—gallic acid, protocatechuic acid, methyl gallate, ethyl gallate, vanillic acid, syringic acid, and quercitrin—in the 70% ethanol extract of PC were determined according to a previously reported method [38], with methodological validation performed in accordance with the Chinese Pharmacopoeia guidelines for analytical method validation [20]. Chromatographic conditions were as follows: The mobile phase consisted of solvent A (water with 0.1% formic acid) and solvent B (acetonitrile with 0.1% formic acid). The gradient elution program was as follows: 0–5 min, 5% B; 5–25 min, 5–15% B; 25–55 min, 15–60% B; 55–60 min, 60–100% B. The flow rate was set at 0.80 mL/min, detection wavelength at 254 nm, column temperature at 40 °C, and injection volume at 10 μL. Separation was achieved on an Inertsil ODS-SP column (4.6 × 150 mm, 5.0 μm). Preparation of standard solutions: Accurately weighed gallic acid, protocatechuic acid, methyl gallate, ethyl gallate, vanillic acid, syringic acid, and quercitrin reference standards were dissolved in methanol and diluted to obtain individual and mixed standard stock solutions. The mixed stock was further serially diluted to prepare a calibration series (1 ng/mL−10 μg/mL). Preparation of sample solutions: An accurately weighed portion of the 70% ethanol extract of PC (0.3 g) was dissolved in 50 mL of 50% methanol, followed by ultrasonic extraction for 1 h. The solution was adjusted for weight loss, diluted 10-fold with 50% methanol, and filtered through a 0.22 μm membrane to yield the test solution. Three parallel samples were prepared. Both standard and sample solutions were analyzed under the same chromatographic conditions. The concentrations of individual compounds were calculated based on calibration curves and dilution factors, and the mass fractions of the seven constituents in the extract were determined. The analytical method was validated for linearity, limit of quantification, precision, accuracy, and stability in compliance with the Chinese Pharmacopoeia guidelines [20]. Detailed procedures and results are provided in the Supplementary Material (Method validation for quantitative analysis in the 70% Ethanol Extract of *Polygonum capitatum*).

### 4.5. Establishment of the AP Rat Model

The rat model of AP was established as previously described [39], with minor modifications. After fasting for 12 h, the rats were anesthetized with an intraperitoneal injection of 3% sodium pentobarbital (40 mg/kg) and placed in the supine position on a surgical table. The abdominal skin was disinfected with povidone–iodine followed by ethanol. A midline lower abdominal incision (~2 cm) was made, and the abdominal wall was carefully opened layer by layer to expose the peritoneal cavity. The left ureter was identified along the posterior abdominal wall. A 4-0 surgical suture was passed through the lateral abdominal wall on either side of the mid-ureter and exteriorized. The penis was temporarily clamped with an arterial clip, and 0.5 mL of *E. coli* suspension (OD600 = 0.35–0.40) was slowly injected into the bladder using a TB syringe. The externalized suture ends were tightened to partially ligate the ureter (approximately one-third of its diameter) and secured on the dorsal side of the rat. The abdominal wall was then closed in layers, and the animals were returned to their cages with free access to food and water. Two hours after surgery, the arterial clip was removed. After 24 h, the exteriorized ureteral ligature was released to restore urinary flow. Rats were allowed to recover for one day before subsequent experimental procedures. In this study, *E. coli* was selected as the model pathogen because it is the most common causative organism in AP and provides a well-established and reproducible in vivo model for pharmacological evaluation.

### 4.6. Pharmacokinetic Study

#### 4.6.1. Animal Grouping and Sample Collection

Rats were randomly assigned to two groups: the normal group and the AP model group (*n* = 8 per group). Beginning on the second day after model induction, rats received the 70% ethanol extract of PC at 0.42 g/kg by oral gavage once daily for 7 consecutive days. This dose was selected with reference to the clinically used crude herb dose, extraction yield, and the preliminary antibacterial and anti-inflammatory activity results described above. After the final administration, blood and renal cortical extracellular fluid microdialysis samples were collected following established protocols [40,41]. Under general anesthesia (induced with 5% isoflurane and maintained with 2.5% isoflurane), rats were placed in the supine position. A heparinized polyethylene catheter was surgically implanted into the right jugular vein for blood collection. A microdialysis probe was implanted into the left renal cortex along the long axis of the kidney (depth: 1–1.5 mm). The probe was perfused with a saline solution at a flow rate of 1 μL/min, followed by a 1 h equilibration period before drug administration. Blood samples (~200 μL) were collected at the following time points: 0, 10, 20, 30, 40, 60, 80, 100, 120, 180, 240, and 300 min for plasma, and at the following intervals for microdialysis samples: -60 to 0 min, 0 to 60 min, 60 to 120 min, 120 to 180 min, 180 to 240 min, and 240 to 300 min.

#### 4.6.2. Sample Preparation

Plasma sample: An aliquot of 50 μL plasma was accurately pipetted into a tube, followed by the addition of 10 μL of internal standard (IS) Bergeninum (1000 ng/mL). Then, 100 μL of 70% acetone in water (containing 0.1% formic acid) was added, and the mixture was vortexed for 1 min. Afterward, 400 μL of ethyl acetate was added and vortexed, and the mixture was centrifuged at 13,000 rpm for 5 min at 4 °C. The supernatant (500 μL) was carefully transferred into a clean EP tube. The residual sample was re-extracted with another 400 μL of ethyl acetate, vortexed, and centrifuged again at 13,000 rpm for 5 min at 4 °C. The supernatant (500 μL) was collected and combined with the previous extract. After solvent evaporation, the residue was reconstituted with 50 μL of 10% acetone in water (containing 0.1% formic acid) for analysis.

Renal cortex microdialysis sample preparation: An aliquot of 50 μL of renal microdialysis sample was pipetted into a clean tube, followed by the addition of 10 μL of IS Bergeninum (1000 ng/mL). The sample was centrifuged at 13,000 rpm for 5 min. The supernatant was transferred into a clean EP tube, and the process was repeated. The final supernatant was isolated for analysis.

#### 4.6.3. Analytical Conditions

Chromatographic separation was performed on an ACQUITY UPLC BEH Shield RP18 column (2.1 mm × 50 mm, 1.7 μm; Waters, USA). The mobile phase consisted of solvent A (water with 0.1% formic acid) and solvent B (methanol with 5 mM ammonium acetate). The gradient elution program was as follows: 0–5 min, 90–70% B; 5–7 min, 70% B; 7–10 min, 70–30% B; 10–12 min, 30–0% B; 12–13 min, 0% B; 13.01–16 min, 90% B. The flow rate was 0.2 mL/min, the column temperature was maintained at 30 °C, and the injection volume was 10 μL.

Mass spectrometric analysis was carried out using an electrospray ionization (ESI) source operated in multiple reaction monitoring (MRM) mode. The optimized MS parameters were as follows: gas temperature, 300 °C; drying gas flow, 7 L/min; nebulizer pressure, 35 psi; sheath gas temperature, 325 °C; sheath gas flow, 12 L/min; capillary voltage, 2000 V; nozzle voltage, 0 V. Analytes included gallic acid, protocatechuic acid, vanillic acid, syringic acid, methyl gallate, ethyl gallate, quercitrin, and the internal standard (IS). Quantification was conducted in the negative ion mode. The optimized MRM transitions and corresponding MS conditions are summarized in Table 4.

#### 4.6.4. Preparation of Calibration Standards and QC Samples

Each reference standard was accurately weighed, dissolved in methanol, and diluted to prepare a mixed stock solution containing gallic acid, protocatechuic acid, vanillic acid, syringic acid, methyl gallate, ethyl gallate, and quercitrin. Serial dilutions of the mixed stock solution were performed with methanol to obtain calibration standards at different concentrations. For QC samples, low-, medium-, and high-concentration solutions were prepared by evaporating the solvent from the respective calibration solutions using a low-temperature concentrator and reconstituting them with blank plasma. The final concentrations of QC samples were as follows: low QC (LQC), 30 ng/mL; medium QC (MQC), 300 ng/mL; high QC (HQC), 1000 ng/mL.

#### 4.6.5. Method Validation Procedure

Method validation for rat plasma analysis was performed in accordance with the guidelines for bioanalytical method validation [20]. The parameters evaluated included specificity, linearity, lower limit of quantification (LLOQ), recovery, matrix effect, precision, accuracy, and stability. Because the biological matrix of the microdialysate samples was simpler than that of plasma, only partial method validation was conducted for the microdialysis samples. Detailed procedures and results are provided in the Supplementary Materials (Pharmacokinetic bioanalytical method validation).

#### 4.6.6. Pharmacokinetic Study in Rats After Oral Administration of the PC Extract

After oral administration of the PC extract, the concentrations of seven compounds in plasma and renal cortical microdialysates were determined using the aforementioned methods. The plasma concentration–time curves for each compound were plotted for both normal and AP rats following drug administration.

Pharmacokinetic parameters were calculated using non-compartmental analysis in WinNonlin software (version 8.1; Certara, USA). The maximum plasma concentration (Cmax) and the time to reach Cmax (Tmax) were obtained from experimental data, while the area under the concentration–time curve (AUC) was calculated using the trapezoidal rule.

### 4.7. Pharmacodynamic Study

Rats were randomly assigned to four groups (*n* = 8 per group): sham group, model group, PC 70% ethanol extract group (PC, 0.42 g/kg), and positive control group (levofloxacin, 79 mg/kg). Oral administration began on the second day after model establishment and continued once daily for seven consecutive days. Rats in the sham and model groups received an equivalent volume of purified water.

#### 4.7.1. Bacteriological Examination

Urine samples collected after the final administration were subjected to bacteriological examination. A 20 μL aliquot of urine from each rat was streaked onto agar plates and incubated for 24 h. One-quarter of the colonies on each plate was scraped and suspended in 25 mL of sterile physiological saline. The optical density (OD) of the suspension was measured at 600 nm, with physiological saline as the blank control. Subsequently, 100 μL of the bacterial suspension was inoculated into Petri dishes containing culture medium and gently mixed, and the dishes were incubated at 37 °C for 24 h in a biochemical incubator. Results were evaluated using a semi-quantitative scoring method: bacterial counts >10,000 were considered positive, whereas counts <500 were considered negative.

#### 4.7.2. Urinalysis

Urine samples were collected on day 7 after administration for routine urinalysis. Rats were placed individually in sterilized metabolic cages, and urine was collected into sterile containers. All samples were analyzed within 1 h at room temperature. Briefly, a reagent strip was immersed in urine for 2 s, excess urine was removed using sterile absorbent paper, and the strip was immediately placed in an automated urine analyzer to obtain results. The measured parameters included urine protein (PRO), occult blood (BLD), ketone bodies (KET), glucose (GLU), bilirubin (BIL), and urobilinogen (URO).

#### 4.7.3. Renal Function Tests

Serum samples were collected after the final administration, and renal function was assessed by measuring Scr and BUN levels. These indicators were used to assess renal filtration and excretory capacity.

#### 4.7.4. Immunological Assays

Urine, serum, and renal tissue were collected after the final administration. The levels of inflammatory cytokines, including TNF-α, IL-2, and MCP-1, were measured using enzyme-linked immunosorbent assay (ELISA) kits, following the manufacturers’ instructions.

#### 4.7.5. Renal Organ Index

Both kidneys were harvested and weighed to calculate the renal organ index and the ratio of left to right kidney mass. The renal index (%) was calculated as: Renal index= kidney weight/body weight ×100%. The kidney mass ratio was calculated as: Left-to-right kidney ratio= left kidney weight/right kidney weight ×100%.

#### 4.7.6. Renal Histopathology

The left kidney was fixed in 10% neutral buffered formalin, embedded in paraffin, and sectioned at a thickness of 5 μm. Sections were stained with hematoxylin for 15 min and eosin for 10 s. After dehydration, clearing, and mounting, the slides were scanned using an automated optical slide scanner. Pathological changes were evaluated and scored based on histopathological analysis.

### 4.8. Correlation Analysis Between Pharmacokinetics and Pharmacodynamics

Plasma samples collected at different time points during the pharmacokinetic experiments were analyzed using ELISA to determine the levels of PRO, BLD, KET, BUN, Scr, GLU, BIL, URO, TNF-α, IL-2, and MCP-1, thereby obtaining pharmacodynamic data.

Robust transformation of pharmacokinetics (PK) and pharmacodynamics (PD) data: To eliminate differences in measurement scales across variables, a robust transformation based on the overall distribution was applied [42]. This transformation preserved the relative order of the data, normalized all values to the (0, 1) interval, and reduced the influence of outliers.

PK–PD correlation analysis: At each time point, the differences in plasma concentrations of a given compound between individuals were used as the independent variable, and the corresponding differences in pharmacodynamic parameters were used as the dependent variable. Specifically, let Yᵢt represent the pharmacodynamic measurement of individual i at time t, and Xᵢt denote the plasma concentration of a given compound for the same individual and time. For any pair of individuals i, j (i, j = 1, 2, …, 6), the following differences were defined:

ΔY t = Yit − Y jt; ΔX t = X it − X jt

Thus, for each compound at each time point t, a total of C _2_^6^ = 15 pairs were generated, with 11 time points yielding 165 PK–PD data pairs for each pharmacodynamic indicator. This increased sample size not only reduced the effect of time-specific variability but also improved the reliability of the analysis.

Spearman’s correlation coefficients between ΔY _t_ and ΔX _t_ were calculated, and a coefficient greater than 0.5 was considered indicative of a relatively strong correlation [43].

### 4.9. Metabolomics Study

#### 4.9.1. Sample Collection and Pretreatment

At the end of the pharmacodynamic study, the remaining portion of the left kidney was excised. All samples were immediately stored at −80 °C until analysis.

Preparation of samples for untargeted metabolomics: Approximately 50 mg of kidney tissue was accurately weighed and homogenized with 400 μL of cold methanol by vortexing for 2 min, followed by grinding with two steel beads at 50 Hz for 4 min at low temperature. The homogenates were kept at −20 °C for 10 min and centrifuged at 14,000 g for 15 min at 4 °C. A 200 μL aliquot of the supernatant was evaporated to dryness at low temperature, reconstituted with 100 μL of methanol/water (*v*/*v*, 1:4), and centrifuged again. The resulting supernatant was used for mass spectrometric analysis.

Preparation of samples for targeted metabolomics: Samples were prepared according to our previously reported method [43]. Briefly, approximately 50 mg of kidney tissue was homogenized in 150 μL of acetonitrile/water (*v*/*v*, 3:1), vortexed at 8000 rpm for 5 min, and centrifuged at 20,000 g for 10 min. A 10 μL aliquot of the supernatant was mixed with 10 μL of 100 mM sodium tetraborate and 10 μL of 1% benzoyl chloride, vortexed for 5 min, and incubated at 25 °C for 5 min, followed by centrifugation at 20,000× *g* for 10 min. Finally, 24 μL of the supernatant was mixed with 6 μL of an internal standard mixture (Trp–Kyn pathway standards derivatized with d5-benzoyl chloride), vortexed, and subjected to mass spectrometric analysis.

#### 4.9.2. Untargeted Metabolomics Analysis Conditions

Untargeted metabolomics was performed using a HPLC-QE-Orbitrap/MS system (Thermo Fisher Scientific, Waltham, Massachusetts, USA). Data were acquired in both positive and negative ion modes, with retention time, MS1, and MS2 spectra collected separately.

Chromatographic conditions were as follows: In positive ion mode, separation was achieved using a Waters BEH C8 column (2.1 × 100 mm, 1.7 μm). The mobile phases consisted of solvent A (water with 0.1% formic acid) and solvent B (acetonitrile with 0.1% formic acid). The gradient program was as follows: 0–1 min, 5% B; 1.1–11 min, 5–100% B; 11.1–13 min, 100% B; 13.1–15 min, 5% B. The flow rate was 0.35 mL·min^−1^, the column temperature was maintained at 50 °C, the autosampler was kept at 4 °C, and the injection volume was 5 μL. For negative ion mode, a Waters HSS T3 column (2.1 × 100 mm, 1.8 μm) was used, while the other conditions remained the same. A pooled QC sample was injected after every ten samples to monitor system stability.

Mass spectrometry conditions: An electrospray ionization (ESI) source was employed in full MS scan combined with data-dependent acquisition (DDA) for MS/MS. Parameters were set as follows: spray voltage, +3.8 kV/−3.0 kV; capillary temperature, 320 °C; auxiliary gas heater temperature, 350 °C; sheath gas flow rate, 35 Arb; auxiliary gas flow rate, 8 Arb; S-lens RF level, 50; mass range, *m*/*z* 70–1050; full MS resolution, 70,000; MS/MS resolution, 17,500; TopN, 5; normalized collision energy (NCE)/stepped NCE, 20 and 40.

#### 4.9.3. Targeted Metabolomics Analysis of the Tryptophan Metabolic Pathway

Targeted metabolomics analysis was performed on an HPLC-MS/MS system.

Chromatographic conditions were as follows: Separation was achieved using a PFP C18 column (2.1 × 50 mm, 1.8 μm; Waters, USA). The mobile phases consisted of solvent A (water containing 5 mM ammonium formate and 0.1% formic acid) and solvent B (acetonitrile). The gradient program was set as follows: 0–1.0 min, 20–20% B; 1.0–2.0 min, 20–50% B; 2.0–6.0 min, 50–70% B; 6.0–6.5 min, 70–95% B; 6.5–8.0 min, 95–95% B; 8.0–8.1 min, 95–20% B; 8.1–10.0 min, 20–20% B. The flow rate was maintained at 0.3 mL·min^−1^, the column temperature at 35 °C, and the autosampler at 4 °C, and the injection volume was 2 μL.

Mass spectrometry conditions: An electrospray ionization (ESI) source was employed with the following parameters: curtain gas (N_2_), 35 psi; collision gas (N_2_), 9 psi; ion spray voltage, 5500 V; ion source temperature, 550 °C; ion source gas 1 (N_2_), 55 psi; ion source gas 2 (N_2_), 55 psi. Data acquisition was performed in multiple reaction monitoring (MRM) mode with positive ion scanning. The ion transition parameters for metabolites in the Trp–Kyn pathway were established based on our previously published method and are detailed in Supplementary Table S1 [43].

#### 4.9.4. Metabolomics Analysis

Mass spectral features were first identified and extracted, followed by peak alignment across multiple samples to generate a unified peak table for multivariate statistical analysis. Principal component analysis (PCA) was applied to provide an overview of the major variation patterns within the dataset, while orthogonal partial least squares-discriminant analysis (OPLS-DA) was employed to identify metabolites that contributed to group discrimination. The quality of the OPLS-DA model was evaluated using the parameters R^2^Y and Q^2^. Metabolites were considered significantly altered when they met the following criteria: VIP > 1, Student’s *t*-test (*p* < 0.05), and fold change (FC) > 1.5 or < 0.7.

Annotation of differential metabolites was performed based on reference standards, Kyoto Encyclopedia of Genes and Genomes (KEGG) target database, integrated multi-source databases, and unknown compound prediction. The annotated metabolites were subsequently mapped to metabolic pathways. By analyzing the number and proportion of these metabolites matched within different pathways, we identified key metabolic pathways that may underlie the biological significance of the observed differences. Data analysis was performed using the One-MAP platform, Metware Cloud platform (https://cloud.metware.cn/ (accessed on 30 April 2026)), and MetaboAnalyst 6.0.

### 4.10. Statistical Analysis

All statistical analyses were performed using SPSS 20.0 and GraphPad Prism 8.0 software. Data are expressed as mean ± standard error of the mean (SEM). Normality was assessed using the Shapiro–Wilk test, and homogeneity of variance was evaluated using Levene’s test. For comparisons between two groups, Student’s *t*-test was used for normally distributed data with equal variances, whereas the Mann–Whitney *U* test was applied for non-normally distributed data. For comparisons among multiple groups, one-way or two-way ANOVA was used as appropriate, followed by suitable post hoc tests.

## 5. Conclusions

In summary, our findings suggest that PC exerts antibacterial and anti-inflammatory effects in AP rats. Gallic acid, protocatechuic acid, methyl gallate, and quercitrin may represent candidate constituents associated with the therapeutic effects of PC. Metabolomic analyses further suggest that disturbances in glycerophospholipid metabolism and the Trp–Kyn pathway may be associated with the response to PC treatment, although these pathway-level findings still require further validation. These results provide preclinical pharmacological evidence supporting the therapeutic potential of PC in bacterial AP and offer a foundation for future investigations into its clinical application. By integrating chemical profiling, exploratory PK–PD analysis, and metabolomics, this study provides preliminary clues to the metabolic pathways potentially associated with the therapeutic effects of PC and may serve as a reference for investigating the pharmacological basis of multi-component traditional medicines.

## Figures and Tables

**Table 1:** Pharmacokinetic parameters of 6 constituents of *Polygonum capitatum* (PC) in plasma from normal rats (left) and AP rats (right) (*n* = 8).

Pharmacokinetic Parameters	Normal						Model					
	Gallic Acid	Protocatechuic Acid	Vanillic Acid	Syringic Acid	Methyl Gallate	Quercitrin	Gallic Acid	Protocatechuic Acid	Vanillic Acid	Syringic Acid	Methyl Gallate	Quercitrin
HL_λz_/min	79.2 ± 27.3	71.5 ± 18.2	264.1 ± 125.7	124.4 ± 58.8	92.3 ± 31.8	57.1 ± 12.1	105.6 ± 71.3	65.9 ± 35.7	108.6 ± 57.5 *	71.9 ± 24.3 *	75.3 ± 48.3	84.6 ± 41.1
T_max_/min	85.0 ± 35.6	61.7 ± 22.3	66.7 ± 24.2	45.0 ± 32.7	116.7 ± 58.5	46.7 ± 26.6	70 ± 42.9	41.7 ± 30.6	46.7 ± 39.8	41.7 ± 33.7	130 ± 45.2	48.3 ± 29.3
C_max_/ng∙mL ^−1^	1046.1 ± 774.8	17.7 ± 9.3	25.8 ± 6.9	26.5 ± 9.3	9.5 ± 5.1	72.3 ± 68.2	1603.3 ± 1026.6	33.9 ± 16.3 *	44.1 ± 11.6	50.1 ± 19.3 *	15.9 ± 8.1	100.5 ± 77.9 *
AUC_last_/ng·mL ^−1^·min	136,855.3 ± 111,662.4	1839.0 ± 1068.5	4460.6 ± 1784.8	3451.4 ± 2305.0	1421.1 ± 826.0	8314.2 ± 10086.1	218,055.2 ± 157,327.5	3060.5 ± 1824.2	4991.6 ± 1230.8	5280.2 ± 1610.7	2363.7 ± 1368.7	8388.4 ± 4523.8
AUC_inf_/ng·mL ^−1^·min	163,964.1 ± 161,935.0	2099.9 ± 1213.1	9650.8 ± 4521.4	4850.6 ± 2201.2	1833 ± 1029.0	9019.1 ± 11,029.4	320,792.8 ± 216,301.5	3469.2 ± 2166.2	6625.3 ± 1920.0	6213.7 ± 1483.4	2745.9 ± 1571.9	9400.1 ± 4251.3
*Vz/F*/mL∙kg ^−1^	15446 ± 11071.2	24301.9 ± 16492.1	13144.2 ± 1173.7	2031.3 ± 1022.6	57702.4 ± 38116.6	8573.1 ± 6031.2	11,322.6 ± 8532.8	12,277.2 ± 7501.2	7440.6 ± 2517.2	825.8 ± 189.1 *	35,400.1 ± 49,093.0	7029.8 ± 3280.2
CL/F/mL∙min∙kg ^−1^	136.4 ± 81.2	233.7 ± 125.0	46.5 ± 35.8,	2.8 ± 7.4	500.1 ± 430.2	105.2 ± 65.2	183.4 ± 291.2	158.1 ± 98.9	54.8 ± 22.6 *	8.5 ± 2.4	279.9 ± 185.7	59.5 ± 24.5
MRT_last_/min	125.7 ± 19.8	104.2 ± 18.1	117.2 ± 35.8	99.1 ± 43.5	138.9 ± 27.3	89.4 ± 21.7	105.6 ± 43.0	83.5 ± 38.5	93.2 ± 32.8	101.4 ± 29.9	128.2 ± 24.3	108.4 ± 21.7

HL_λz_, terminal half-life; λz, first-order rate constant associated with the terminal (log-linear) portion of the curve, estimated by linear regression of time vs. log concentration; T_max_, time to C_max_; C_max_, maximum plasma concentration; AUC_last_, area under the curve; AUC_inf_, AUC from dosing time extrapolated to infinity, based on the last observed concentration; *V_z_*/*F*, dose/(λz∙AUC_inf_); CL/F, Dose/AUC_inf_; MRT_last_, residence time from the time of dosing to the time of the last measurable concentration. * *p* < 0.05 (compared to the normal rats).

**Table 2:** Pharmacokinetic parameters of 6 constituents of *Polygonum capitatum* (PC) in the renal cortex of normal rats (left) and AP rats (right) (*n* = 8). * *p* < 0.05 (compared to the normal rats).

Pharmacokinetic Parameters	Normal				Model			
	Gallic Acid	Protocatechuic Acid	Methyl Gallate	Quercitrin	Gallic Acid	Protocatechuic Acid	Methyl Gallate	Quercitrin
HL_λz_/min	233.6 ± 160.0	329.1 ± 425.8	192.1 ± 138.4	97.7 ± 61.2	197.2 ± 127.1	278.3 ± 392.1	592.1 ± 1067.1	80.9 ± 70.7
T_max_/min	150 ± 60.0	162.9 ± 82.8	205.7 ± 76.3	111.4 ± 41.4	180 ± 103.9	168 ± 78.2	200 ± 72.7	210 ± 103.9 *
C_max_/ng∙mL ^−1^	521.5 ± 153.6	25.3 ± 16.8	14.8 ± 7.7	80.8 ± 87	466.3 ± 262.4	34.8 ± 32.9	11.9 ± 8.9	101.4 ± 90.4
AUC_last_/ng·mL ^−1^·min	104,955.1 ± 24,776.9	3779.6 ± 1462.6	2337.1 ± 1622.2	11,342.0 ± 7295.8	87,660.8 ± 40,095.0	5886.7 ± 4645.0	2133.8 ± 1458.8	12,846.1 ± 10,503.7
MRT_last_/min	167.2 ± 21.2	159.2 ± 26.4	186.2 ± 24.9	139.1 ± 19.1	164.4 ± 13.7	157.1 ± 24.7	180.8 ± 19.5	154 ± 49.3

**Table 3:** Correlation analysis between pharmacological parameters and major components in *Polygonum capitatum* (PC).

Efficacy Index		Correlation Coefficient						
		Gallic Acid	Protocatechuic Acid	Vanillic Acid	Syringic Acid	Ethyl Gallate	Methyl Gallate	Quercitrin
1	PRO	0.514	0.664	0.445	0.147	0.177	0.174	0.189
2	KET	0.265	0.548	0.632	0.355	0.438	0.168	0.565
3	TNF-α	0.733	0.314	0.653	0.378	0.372	0.385	0.211
4	IL-2	0.467	0.748	0.233	0.533	0.689	0.812	0.346
5	MCP-1	0.688	0.552	0.458	0.469	0.243	0.539	0.314

**Table 4:** MRM transitions and MS conditions for quantification of seven analytes and internal standard.

Compound	Parent Ion (*m*/*z*)	Product Ion (*m*/*z*)	Fragmentor	CE
Gallic acid	169	125	80	10
Protocatechuic acid	153	109	80	10
Vanillic acid	167	152	100	5
Syringic acid	197	182	100	5
Methyl gallate	183	124	100	20
Ethyl gallate	197	169	100	10
Quercitrin	447.2	300.1	160	20
Is-Bergeninum	327	312	100	10

## Data Availability

The data reported in this paper have been deposited in the OMIX, China National Center for Bioinformation/Beijing Institute of Genomics, Chinese Academy of Sciences (https://ngdc.cncb.ac.cn/omix: accession no.OMIX013477 (accessed on 30 April 2026)).

## Supplementary Materials

The following supporting information can be downloaded at: https://www.mdpi.com/article/10.3390/ijms27104399/s1.

## Abbreviations

The following abbreviations are used in this manuscript:

AP	Acute pyelonephritis
BIL	Bilirubin
BLD	Occult blood
BUN	Blood urea nitrogen
CL	Cardiolipin
ESI	Electrospray ionization
GLU	Glucose
HPLC	High-performance liquid chromatography
HPLC-MS/MS	High-performance liquid chromatography–tandem mass spectrometry
MRM	Multiple reaction monitoring
PD	Pharmacodynamics
PK	Pharmacokinetics
UTI	Urinary tract infection

## References

[1] Mancuso G., Midiri A., Gerace E., Marra M., Zummo S., Biondo C. (2023). Urinary Tract Infections: The Current Scenario and Future Prospects. Pathogens.

[2] Advani S.D., Thaden J.T., Perez R., Stair S.L., Lee U.J., Siddiqui N.Y. (2025). State-of-the-Art Review: Recurrent Uncomplicated Urinary Tract Infections in Women. Clin. Infect. Dis..

[3] Schwartz L., De Dios Ruiz-Rosado J., Stonebrook E., Becknell B., Spencer J.D. (2023). Uropathogen and Host Responses in Pyelonephritis. Nat. Rev. Nephrol..

[4] Cetin N., Kiraz Z.K., Gencler A. (2020). Serum Presepsin, Proadrenomedullin and Triggering Receptor Expressed on Myeloid Cells-1 (TREM-1) as Biomarkers for the Diagnosis of Acute Pyelonephritis. Indian Pediatr..

[5] Han B., Yu M., Xiong X.-S. (2023). Expression of Sirt6 in Serum of Patients with Diabetic Nephropathy and its Correlation with Renal Function Indexes and Changes in Intestinal Flora Distribution. J. Parasit. Biol..

[6] Hudson C., Mortimore G. (2020). The Diagnosis and Management of a Patient with Acute Pyelonephritis. Br. J. Nurs..

[7] Hyun M., Lee J.Y., Lim K.R., Kim H.A. (2024). Clinical Characteristics of Uncomplicated Acute Pyelonephritis Caused by *Escherichia coli* and *Klebsiella pneumoniae*. Infect. Dis. Ther..

[8] Ainiwaer P., An J., Lou Y., Zhu J.-R. (2025). Research Progress on Chemical Constituents and Pharmacological Activities of *Brassica rapa* L. Nat. Prod. Res. Dev..

[9] Fan J.-D., Qian Z.-Y., Long Y.-G., Qin R.-G. (2020). Study on the Active Parts of *Peristrophe japonica* of Bacterial Vaginitis Treatment Effect. Guangzhou Med. J..

[10] Huang Y., Sun H.-Y., Qin X.-L., Li Y.-J., Liao S.-G., Gong Z.-P., Lu Y., Wang Y.-L., Wang A.-M., Lan Y.-Y. (2017). A UPLC-MS/MS Method for Simultaneous Determination of Free and Total Forms of a Phenolic Acid and Two Flavonoids in Rat Plasma and Its Application to Comparative Pharmacokinetic Studies of *Polygonum capitatum* Extract in Rats. Molecules.

[11] Liu Q.-Q., Liang X.-L., Li W.-L., Chen Y., Liu W., Huang Z., Li Y., Zeng S., Wei Z. (2021). Research Status of Ethnic Drug *Ventilago leiocarpa* Benth. Hubei Agric. Sci..

[12] Lin Y., He L., Chen X.-J., Zhang X., Yan X.-L., Tu B., Zeng Z., He M.-H. (2022). Polygonum Capitatum, the Hmong Medicinal Flora: A Comprehensive Review of Its Phytochemical, Pharmacological and Pharmacokinetic Characteristics. Molecules.

[13] Fan S., Huang Y., Zuo X., Li Z., Zhang L., Tang J., Lu L., Huang Y. (2022). Exploring the Molecular Mechanism of Action of *Polygonum capitatum* Buch-Ham. Ex D. Don for the Treatment of Bacterial Prostatitis Based on Network Pharmacology and Experimental Verification. J. Ethnopharmacol..

[14] Ma F.-W., Deng Q.-F., Zhou X., Gong X.-J., Zhao Y., Chen H.-G., Zhao C. (2016). The Tissue Distribution and Urinary Excretion Study of Gallic Acid and Protocatechuic Acid after Oral Administration of Polygonum Capitatum Extract in Rats. Molecules.

[15] Wang Z., Jiang X. (2018). Flavonoid-Rich Extract of *Polygonum capitatum* Attenuates High-Fat Diet–Induced Atherosclerosis Development and Inflammatory and Oxidative Stress in Hyperlipidemia Rats. Eur. J. Inflamm..

[16] Jiang Q., Cheng H.-M., Bao G., Su M.-K. (2023). Etiology, Expressions of PCT and IL-6 and Liver and Kidney Function of Patients with Urinary Tract Infection Treated with Relinqing Granules and Cefoxitin. Chin. Arch. Tradit. Chin. Med..

[17] Yan X.-Y., Xiang P., Yu Z.-G., Yan H. (2022). Application of Metabonomics in Substance Abuse Toxicology Research. Fa Yi Xue Za Zhi.

[18] Lei C., Chen Z., Fan L., Xue Z., Chen J., Wang X., Huang Z., Men Y., Yu M., Liu Y. (2022). Integrating Metabolomics and Network Analysis for Exploring the Mechanism Underlying the Antidepressant Activity of Paeoniflorin in Rats With CUMS-Induced Depression. Front. Pharmacol..

[19] Tan B., Zhang Z.-J., Xu F.-G., Zhang P. (2021). Crosstalk between Tryptophan Metabolism and Kidney Disease, Mechanisms and Therapeutic Implications. J. Pharm. Res..

[20] (2020). Chinese Pharmacopoeia Commission Guiding Principles for Quantitative Analysis of Biological Samples. Chinese Pharmacopoeia.

[21] Liao S.-G., Zhang L.-J., Sun F., Zhang J.-J., Chen A.-Y., Lan Y.-Y., Li Y.-J., Wang A.-M., He X., Xiong Y. (2011). Antibacterial and Anti-Inflammatory Effects of Extracts and Fractions from *Polygonum capitatum*. J. Ethnopharmacol..

[22] Yan X.-L., Li C.-Q., Liu Y.-X., Chang X., Kang W.-Y. (2010). Antioxidant Activity of *Polygonum capitatum*. China Pharm..

[23] Yao J., Yang Y.-K., Yang L., Liu C., Ni Y., Li A.-P., Hao X.-L. (2017). Investigation of the Effects and Mechanisms of Hematuria Capsules on Rats with Acute Pyelonephritis Using Cell Regulatory Factors. Chin. Tradit. Pat. Med..

[24] Zhang H. (2020). An Integrative Metabolomics and Network Pharmacology Method for Exploring the Effect and Mechanism of *Radix bupleuri* and *Radix paeoniae* Alba on Anti-Depression. J. Pharm. Biomed. Anal..

[25] Abbas A.K., Trotta E., Simeonov D.R., Marson A., Bluestone J.A. (2018). Revisiting IL-2: Biology and Therapeutic Prospects. Sci. Immunol..

[26] Mizui M. (2019). Natural and Modified IL-2 for the Treatment of Cancer and Autoimmune Diseases. Clin. Immunol..

[27] Charton F., Conan P.L., Le Floch H., Bylicki O., Gaspard W., Soler C., Margery J., Rivière F. (2020). Evaluation of Pneumococcal Urinary Antigen Testing for Respiratory Tract Infection Investigations. Médecine Mal. Infect..

[28] Sahuquillo-Torralba A., Carretero G., Rivera R., Ferrándiz C., Daudén-Tello E., De La Cueva P., Gómez-García F.J., Belinchón I., Herrera-Acosta E., Ruiz-Genao D. (2020). The Risk of Urinary Tract Infections in Patients with Psoriasis on Systemic Medications in Biobadaderm Registry: A Prospective Cohort Study. J. Am. Acad. Dermatol..

[29] Qu L., Jiao B. (2023). The Interplay between Immune and Metabolic Pathways in Kidney Disease. Cells.

[30] Wang L.-M., Wang P., Teka T., Zhang Y.-C., Yang W.-Z., Zhang Y., Wang T., Liu L.-X., Han L.-F., Liu C.-X. (2020). ^1^ H NMR and UHPLC/Q-Orbitrap-MS-Based Metabolomics Combined with 16S rRNA Gut Microbiota Analysis Revealed the Potential Regulation Mechanism of Nuciferine in Hyperuricemia Rats. J. Agric. Food Chem..

[31] Qin N., Jiang Y., Shi W., Wang L., Kong L., Wang C., Guo Y., Zhang J., Ma Q. (2021). High-Throughput Untargeted Serum Metabolomics Analysis of Hyperuricemia Patients by UPLC-Q-TOF/MS. Evid.-Based Complement. Altern. Med..

[32] Zhou J.-B., Zhang X.-M., Wu Z., Wu Y.-S., Gao J.-D. (2023). Study on Shizhifang Ameliorating Pyroptosis of Renal Tubular Epithelial Cells Induced by Hyperuricemia Through Inhibiting NLRP3 Inflammasome. China J. Tradit. Chin. Med. Pharm..

[33] Zhang J., Liu Y., Zhi X., Xu L., Tao J., Cui D., Liu T.F. (2024). Tryptophan Catabolism via the Kynurenine Pathway Regulates Infection and Inflammation: From Mechanisms to Biomarkers and Therapies. Inflamm. Res..

[34] Yu K., Li Q., Sun X., Peng X., Tang Q., Chu H., Zhou L., Wang B., Zhou Z., Deng X. (2023). Bacterial Indole-3-Lactic Acid Affects Epithelium–Macrophage Crosstalk to Regulate Intestinal Homeostasis. Proc. Natl. Acad. Sci. USA.

[35] Hou W., Li S., Wu Y., Du X., Yuan F. (2009). Inhibition of Indoleamine 2, 3-Dioxygenase-Mediated Tryptophan Catabolism Accelerates Crescentic Glomerulonephritis. Clin. Exp. Immunol..

[36] Aregger F., Uehlinger D.E., Fusch G., Bahonjic A., Pschowski R., Walter M., Schefold J.C. (2018). Increased Urinary Excretion of Kynurenic Acid Is Associated with Non-Recovery from Acute Kidney Injury in Critically Ill Patients. BMC Nephrol..

[37] Xing W.-S., Li Y.-H., Zhu Y.-H., Wang Q.-X. (2013). Anti-Inflammatory and Anti-Bacterial Activities of Extractsfrom Mussaenda Parviflora Extract in Animal Models. Chin. J. Exp. Tradit. Med. Formulae.

[38] Zhang K.-X., Wang Y.-S., Jing W.-G., Zhang J., Liu A. (2013). Improved Quality Control Method for Prescriptions of *Polygonum capitatum* through Simultaneous Determination of Nine Major Constituents by HPLC Coupled with Triple Quadruple Mass Spectrometry. Molecules.

[39] Xue W.-Y., Qi J.-C., Du L. (2017). Intervention Effect and Mechanism of Curcumin in Chronic Urinary Tract Infection in Rats. Asian Pac. J. Trop. Med..

[40] Li T., Feng X., Feng X., Peng J., Zhao X.-L., Li J., Yang W.-P. (2021). Development of a Liquid-Liquid Microextraction GC-MS method for Simultaneous Determination and Pharmacokinetic Analysis of β-Elemene in Rat Plasma after Administration of Citronella Grass Extract. Acta Pharm. Sin..

[41] Li T., Zhao X.-L., Gao T.-L., Jiao Y., Gao W.-Y., Liu Y., Zhang M.-Y., Wang Z.-G., Wang D.-Q. (2020). Microdialysis Sampling and HPLC-MS/MS Quantification of Sinomenine, Ligustrazine, Gabapentin, Paracetamol, Pregabalin and Amitriptyline in Rat Blood and Brain Extracellular Fluid. Acta Pharm. Sin..

[42] Lin L., Liu J.-X., Zhang Y., Li X.-Z., Lin C.-R., An J.-B. (2012). A PK/PD Data Processing Study on Chinese Formulation of Shuang-Shen-Tong-Guan Prescription. World Sci. Technol.-Mod. Tradit. Chin. Med..

[43] Yuan Z., Zhao X., Zhang Y., Jiao Y., Liu Y., Gao C., Zhang J., Ma Y., Wang Z., Li T. (2025). Using Integrated Network Pharmacology and Metabolomics to Reveal the Mechanisms of the Combined Intervention of Ligustrazine and Sinomenine in CCI-Induced Neuropathic Pain Rats. Int. J. Mol. Sci..

