# Supplementary material for: Integrative Pharmacokinetic and Metabolomic Profiling of Polygonum capitatum Extract Reveals Renoprotective Mechanisms in a Rat Model of Acute Pyelonephritis
Source: Int J Mol Sci. 2026 May 14;27(10):4399. doi: 10.3390/ijms27104399 (PMC13207366; doi:10.3390/ijms27104399)
Supplement: Supplementary file 1 [file ijms-27-04399-s001.zip › supplementary material/supplementary material.pdf]

Supplementary Data

Supplementary: Method validation for quantitative analysis in the 70% ethanol extract of polygonum capitatum.

Supplementary: Quantitative methodology validation results.

Supplementary: Pharmacokinetic bioanalytical method validation.

Supplementary: Pharmacokinetic methodology validation results.

Supplementary Figure S1-S2

Supplementary Table S1-S5

Table S1. Labeling patterns and MRM conditions for metabolites in the tryptophan pathway

<table>
<tr><th>Metabolic pathways</th><th>Metabolites</th><th>Abbreviation</th><th>Labeling Pattern</th><th>Ionization model</th><th>Transition</th><th>Declustering potential (V)</th><th>Collision energy (eV)</th><th>Retention time (min)</th></tr>
<tr><td>Tryptophan</td><td>Tryptophan</td><td>Trp</td><td>mono</td><td>[M + H]$^+$</td><td>309.2 / 105.1</td><td>100</td><td>35</td><td>3.51</td></tr>
<tr><td rowspan="7">Tryptophan-Serotonin pathway</td><td>5-Hydroxytryptophan</td><td>5-HTP</td><td>bi</td><td>[M + H]$^+$</td><td>429.2 / 105.1</td><td>80</td><td>41</td><td>4.00</td></tr>
<tr><td>Serotonin</td><td>5-HT</td><td>bi</td><td>[M + H]+</td><td>385.1 / 105.1</td><td>45</td><td>38</td><td>4.52</td></tr>
<tr><td>5-Hydroxyindoleacetic acid</td><td>5-HIAA</td><td>mono</td><td>[M + NH_4]$^+$</td><td>313.1 / 146.1</td><td>70</td><td rowspan="3">15</td><td>3.78</td></tr>
<tr><td>5-Hydroxytryptophol</td><td>5-HTOL</td><td>mono</td><td>[M + H]$^+$</td><td>282.1 /160.1</td><td>70</td><td>3.86</td></tr>
<tr><td>N-Acetylserotonin</td><td>NAS</td><td>mono</td><td>[M + H]$^+$</td><td>323.1 /264.1</td><td>70</td><td>3.92</td></tr>
<tr><td>Melatonin</td><td>Mel</td><td>non</td><td>[M + H]$^+$</td><td>233.2 / 174.1</td><td>80</td><td rowspan="2">40</td><td>3.17</td></tr>
<tr><td>4-Aminobenzoic acid</td><td>4-AA</td><td>mono</td><td>[M + H]$^+$</td><td>243.2/105.1</td><td>80</td><td>3.45</td></tr>
<tr><td rowspan="7">Tryptophan-Kynurenine pathway</td><td>Kynurenine</td><td>Kyn</td><td>bi</td><td>[M + H]$^+$</td><td>417.2 / 122.1</td><td>80</td><td>20</td><td>3.98</td></tr>
<tr><td>Kynurenic acid</td><td>KA</td><td>mono</td><td>[M + H]$^+$</td><td>294.2 / 105.1</td><td>80</td><td rowspan="2">40</td><td>4.06</td></tr>
<tr><td>3-Hydroxykynurenine</td><td>3-HK</td><td>tri</td><td>[M + H]$^+$</td><td>537.2 / 240.1</td><td>80</td><td>4.09</td></tr>
<tr><td>3-Hydroxyanthranilic acid</td><td>3-HAA</td><td>bi</td><td>[M + H]$^+$</td><td>362.2 / 240.1</td><td>80</td><td>20</td><td>4.06</td></tr>
<tr><td>Xanthurenic acid</td><td>XA</td><td>bi</td><td>[M + H]$^+$</td><td>414.2 / 105.1</td><td>80</td><td rowspan="2">40</td><td>4.06</td></tr>
<tr><td>Quinolinic acid</td><td>QA</td><td>non</td><td>[M + H]$^+$</td><td>168.2 / 78.1</td><td>40</td><td>0.46</td></tr>
<tr><td>Picolinic acid</td><td>PA</td><td>non</td><td>[M + H]$^+$</td><td>124.1/106.1</td><td>40</td><td>20</td><td>0.45</td></tr>
<tr><td>Tryptophan-Indole pathway</td><td>Tryptamine</td><td>TrpA</td><td>mono</td><td>[M + H]$^+$</td><td>265.2 / 144.1</td><td>50</td><td>19</td><td>3.90</td></tr>
</table>

Method validation for quantitative analysis in the 70% ethanol extract of polygonum capitatum

Precision, repeatability, and stability: Seven standard solutions containing internal standards were prepared and analyzed in six replicates over three consecutive days to determine intra-day and inter-day precision. Repeatability was evaluated by preparing six independent samples following the procedure described in the "Preparation of Sample Solutions" section, which were then analyzed consecutively. Sample stability was assessed by analyzing samples at predetermined time intervals (1, 2, 4, 8, 12, 24, 36, and 48 hours).

Recovery rate: Known amounts of PC 70% ethanol extract were spiked with high, medium, and low concentrations of mixed standard solutions. Each concentration level was analyzed in triplicate, and the recovery rate was calculated as the mean of the three replicates.

Quantitative methodology validation results

Precision, repeatability, and stability: The RSD values were all below 4%, indicating that the instrument exhibited good precision, the preparation method showed good repeatability, and the 70% ethanol extract of PC demonstrated good stability over 48 hours.

Recovery Rate: The average recovery rates of all substances were between 95% and 105%, with RSD values all below 4%, indicating that the method is within the range of system errors and can be used for the determination of the content of PC 70% ethanol extract.

Pharmacokinetic bioanalytical method validation

Specificity: Rat blank plasma samples were processed according to the method outlined under "Plasma sample preparation," and chromatograms were obtained. A certain concentration of mixed reference standard solution was added to rat blank plasma, and chromatograms were obtained following the same procedure. Plasma samples from rats administered Polygonum capitatum extract were processed in the same manner to examine the specificity of the analytical method.

Linear Range and Quantification Limit: Rat blank plasma was spiked with varying concentrations of mixed reference standard solutions to prepare simulated biological samples with concentrations of 1, 2, 5, 10, 20, 50, 100, 200, 500, and 1000 $ng \cdot ml^{-1}$ for each component to be tested. The plasma samples were processed as described under "Plasma sample preparation." The peak area ratio of the reference standard to the internal standard was plotted against the ratio of the reference standard mass concentration to the internal standard mass concentration to construct the standard curve, linear range, and correlation coefficient. The lowest quantifiable limit for each component was calculated based on S/N = 10.

Recovery Rate, Matrix Effect, Precision, and Accuracy: QC samples at low, medium, and high concentrations were prepared in quintuplicate. The plasma samples were processed according to the method outlined under "Plasma sample preparation," and the chromatographic peak areas (A) for each component were recorded. Blank plasma samples were processed as described under "Plasma sample preparation." The supernatant, after protein precipitation, was spiked with the same concentrations of mixed reference standard solution, followed by the same procedure, and chromatographic peak areas (B) were recorded. Additionally, the same concentrations of mixed reference standard solution were processed as described in "Plasma sample preparation" under the method for solvent evaporation in the low-temperature concentrator, and chromatographic peak areas (C) were recorded. The ratio of A to B indicates the extraction recovery rate, and the ratio of B to C represents the matrix effect. The relative recovery rate and accuracy were calculated by comparing the concentration determined from the standard curve with the spiked concentration. The intra-day and inter-day precision and accuracy were determined by measuring QC samples five times within one day and five consecutive days.

Stability: Stability was assessed by subjecting the samples to two freeze-thaw cycles (-80°C for 24 hours, thawed at room temperature). Room temperature stability was assessed by leaving the samples at room temperature for 4 hours. After processing, samples were stored at room temperature for 0, 12, and 24 hours before analysis to assess post-preparation stability. Three replicates were prepared for each condition.

Pharmacokinetic methodology validation results

Linear Range and Limit of Quantification: The typical calibration curve regression equations, coefficients of determination (R^2), and limits of quantification (LLOQ) for each analyte in plasma and microdialysateare presented in Table S2.

Table S2. Calibration curves and parameters of seven analytes in plasma and microdialysate following administration of Polygonum capitatum extract in rats.

Constituents	Regression equation	R^2	LLOQ/ng mL^{-1}
Gallic acid	y=5.03x+0.01	0.992	1.2
Protocatechuic acid	y=23.52x+0.12	0.990	2.1
Vanillic acid	y=0.86x+0.02	0.990	1.6
Syringic acid	y=1.13x+0.002	0.994	2.6
Ethy gallate	y=31.48x+0.08	0.991	1.5
Methy gallate	y=41.75x+0.08	0.995	5.6
Quercitrin	y=2.98x+0.01	0.995	5.1

Recovery, Precision, and Accuracy： The extraction recovery results are summarized in Table 3. At low, medium, and high concentrations, the recovery rates of the seven analytes ranged from 68.9% to 90.2%, with RSDs below 11.5%. No significant matrix effects were observed (82.3%–117.2%), indicating that the sample preparation method was generally applicable for all eight analytes. Both intra- and inter-day precision assays showed that the accuracy of the three concentration levels ranged from 85.3% to 115.5% of the nominal values, with RSDs below 15%, confirming the robustness and feasibility of the analytical method.

Stability： The analytes in plasma exhibited acceptable stability under various conditions. After storage at room temperature for 4 h, relative recoveries (RR) ranged from 80.5% to 115.6%. Following two freeze–thaw cycles (−80 °C/24 h, thawed at room temperature), the RR values ranged from 82.4% to 110.0%. Processed samples stored in the autosampler for 24 h showed RSDs below 12.5%. These results indicate that the analytes were generally stable under the tested conditions, although long-term storage should be avoided.

Fig. S1 Specificity assessment of seven analytes. (A) Gallic acid; (B) Protocatechuic acid; (C) Ethyl gallate; (D) Methyl gallate; (E) Syringic acid; (F) Vanillic acid; (G) Quercitrin. Representative MS/MS spectra of the quantifier ions in blank plasma, QC samples, and plasma samples. From top to bottom: blank plasma; plasma sample from AP model rats; plasma sample from normal rats; IS; QC sample.

Table S3 Effects of PC extract on urinary bacterial culture results in AP rats. ($n=8$, $X \pm S$)

Group	Positive	Negative	Positive rate（%）
Sham	0	8	0
Model	8	0	100$^{##}$
PC	3	5	37.5*
Positive	2	6	25*

$^{##}p < 0.01$ (compared to the Sham group). $^{*}p < 0.05$ (compared to the Model group).

Table S4 Numbers and percentages of rats with positive urinary PRO results in different groups. *($n = 8$)*

Day	Sham		Model		PC		Positive	
	Positive (n)	Positive rate (%)	Positive (n)	Positive rate (%)	Positive (n)	Positive rate (%)	Positive (n)	Positive rate (%)
1	1	12.5	6	75$^{###}$	5	62.5	7	87.5
3	1	12.5	6	75$^{###}$	3	37.5*	3	37.5*
7	0	0	5	62.5$^{###}$	3	37.5*	3	37.5*

$^{###}p < 0.001$ (compared to the Sham group). $^{*}p < 0.05$ (compared to the Model group).

Table S5 Effects of PC on kidney weights and left-to-right kidney weight ratio in AP rats ($n=8$, $\bar{X} \pm S$)

Group	Body weight（g)	Left kidney weight(g)	Right kidney weight (g)	Left/Right kidney ration
Sham	286.72±10.63	1.22±0.14	1.2±0.14	1.01±0.05
Model	275.03±21.45	2.28±1.08	1.31±0.14	1.69±0.69$^{##}$
PC	257.61±26.21	2.11±1.33	1.26±0.17	1.65±1
Positive	274.94±21.72	1.75±0.87	1.33±0.15	1.25±0.52*

$^{##}p < 0.01$ (compared to the Sham group). $^{*}p < 0.05$ (compared to the Model group).

Fig. S2 Total ion chromatograms (TICs) of kidney tissue samples in the untargeted metabolomics analysis. (A) Positive ion mode (B) Negative ion mode
