# Supplementary figures and images for: Integrative Pharmacokinetic and Metabolomic Profiling of Polygonum capitatum Extract Reveals Renoprotective Mechanisms in a Rat Model of Acute Pyelonephritis
Source: Int J Mol Sci. 2026 May 14;27(10):4399. doi: 10.3390/ijms27104399 (PMC13207366; doi:10.3390/ijms27104399)

**A**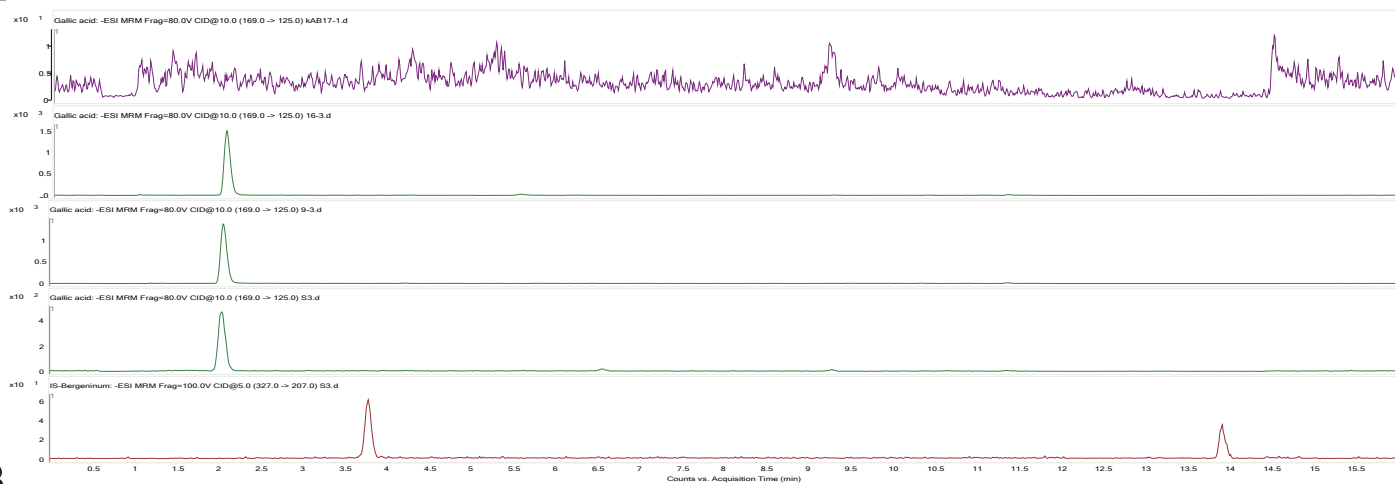**B**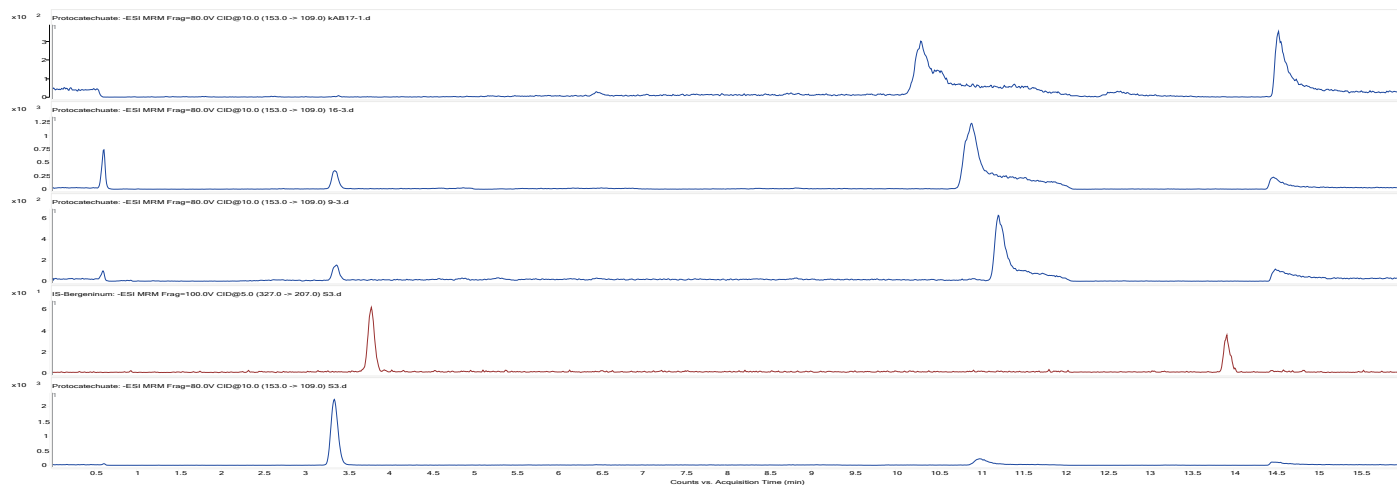**C**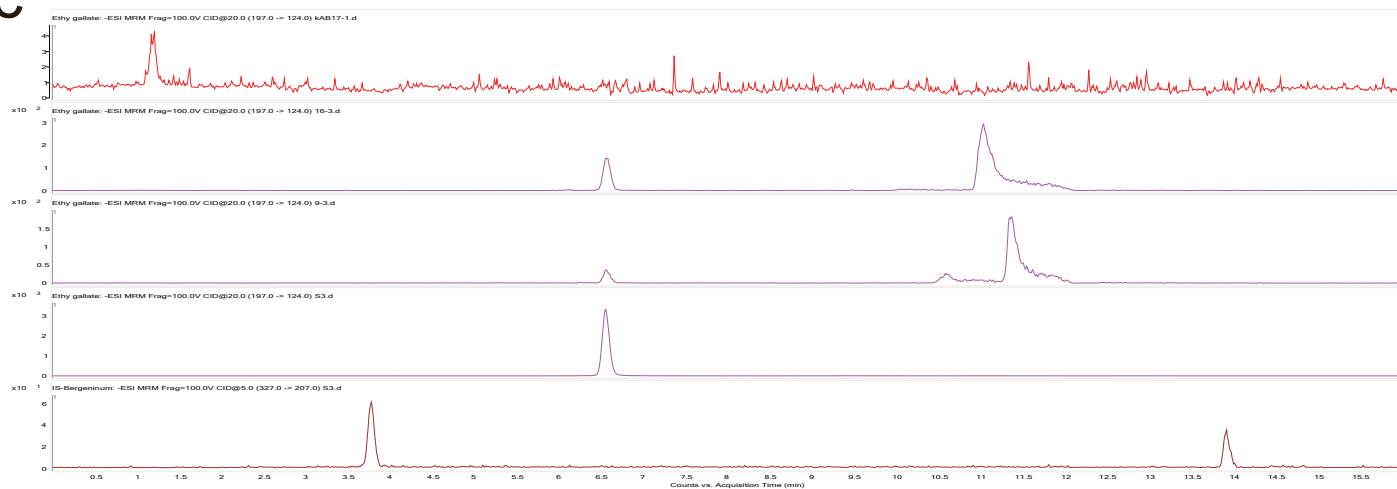**D**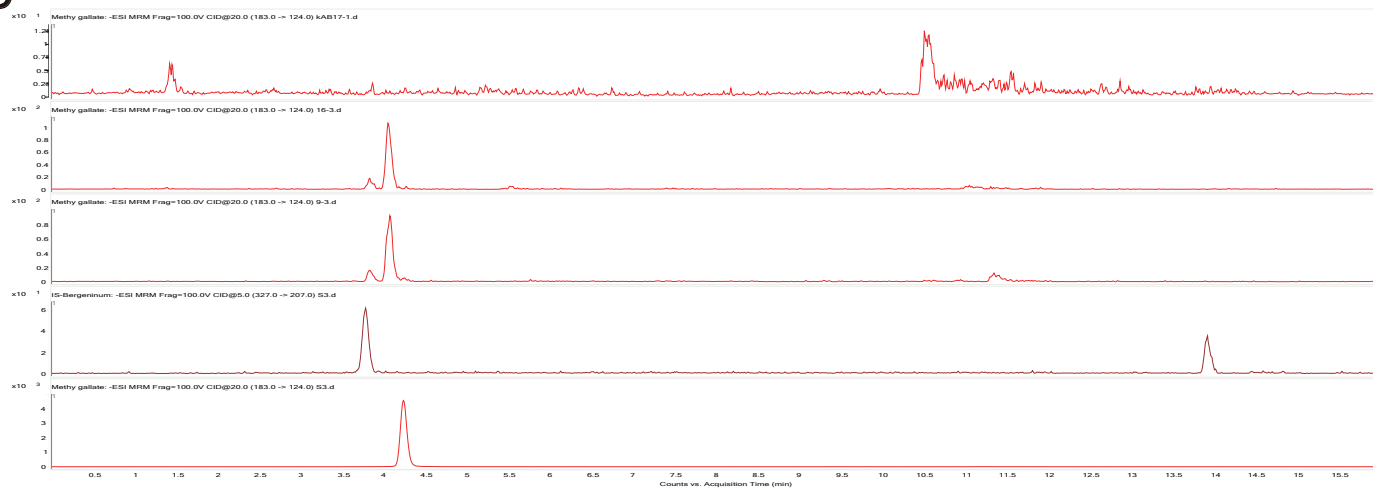

Supplement: Supplementary file 1 [file ijms-27-04399-s001.zip › supplementary material/Fig. S1-1.pdf]

E

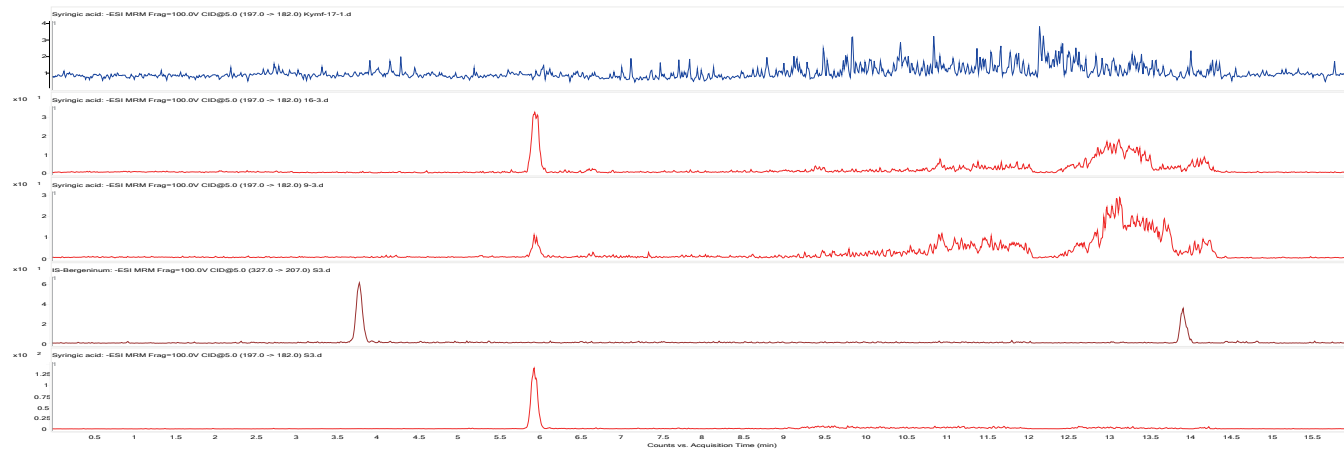

F

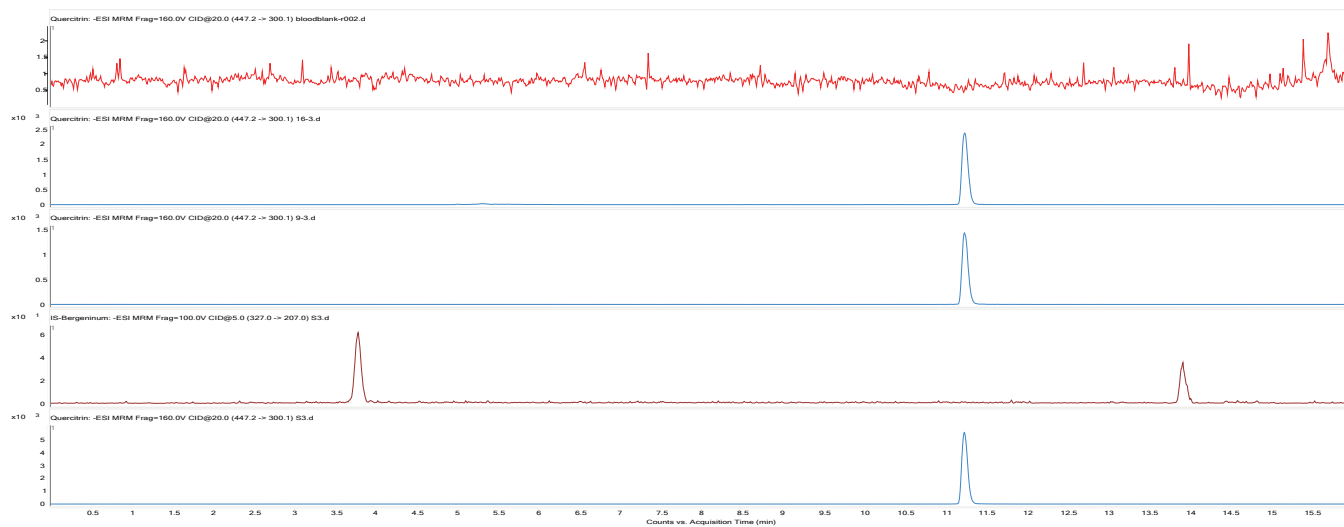

G

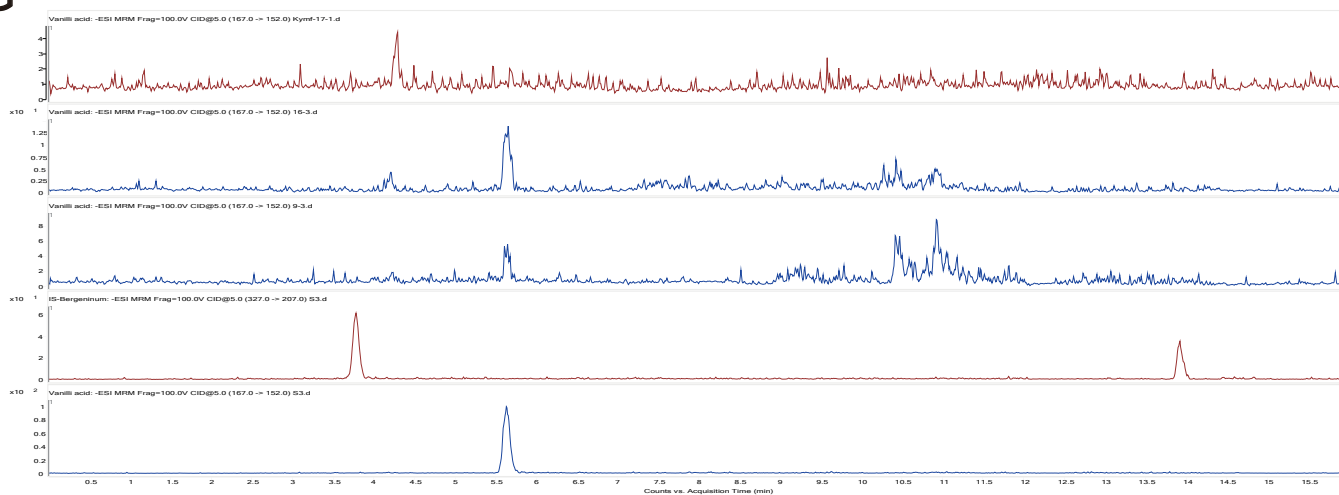

Supplement: Supplementary file 1 [file ijms-27-04399-s001.zip › supplementary material/Fig. S1-2.pdf]

A

XIC, m/z: 69.3061 - 1060.6111

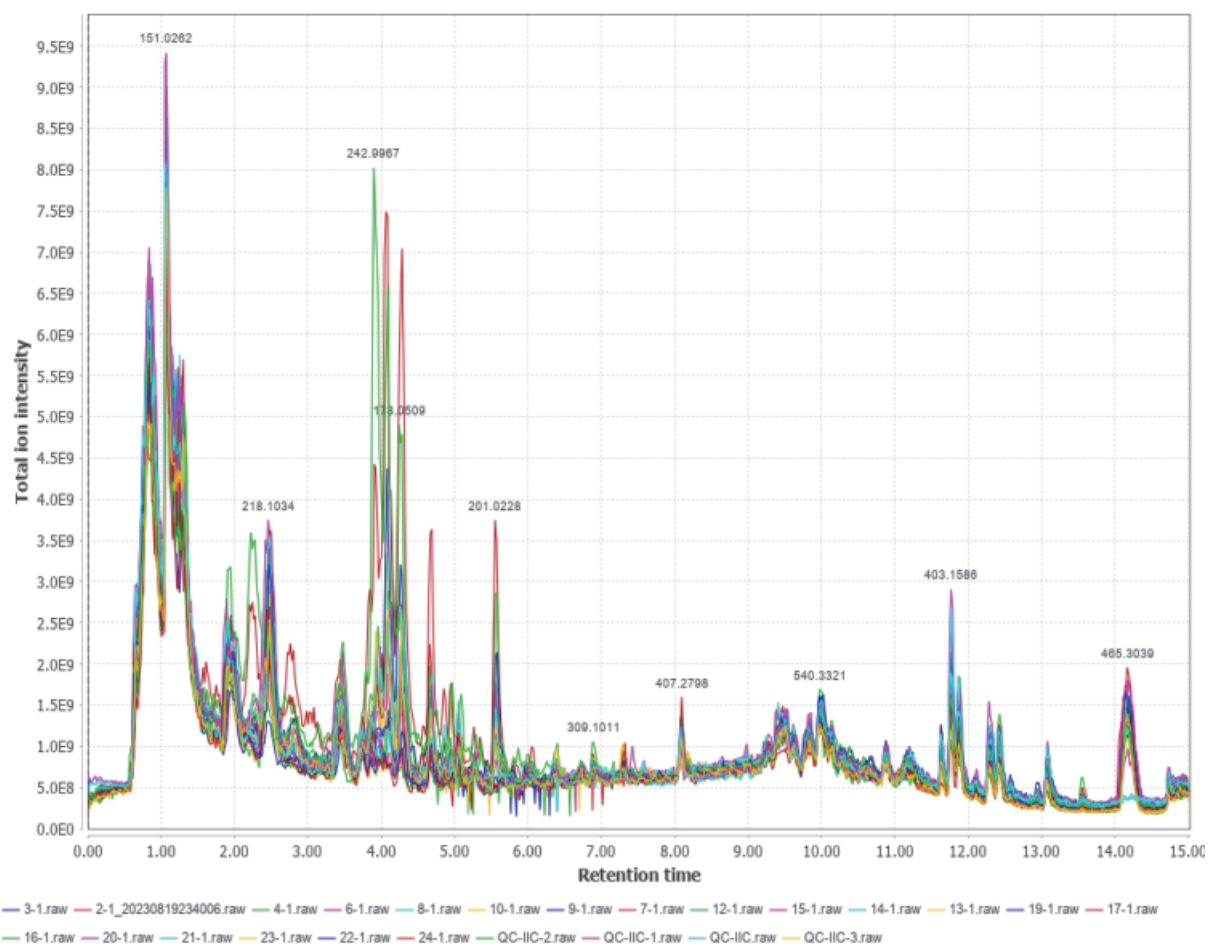

B

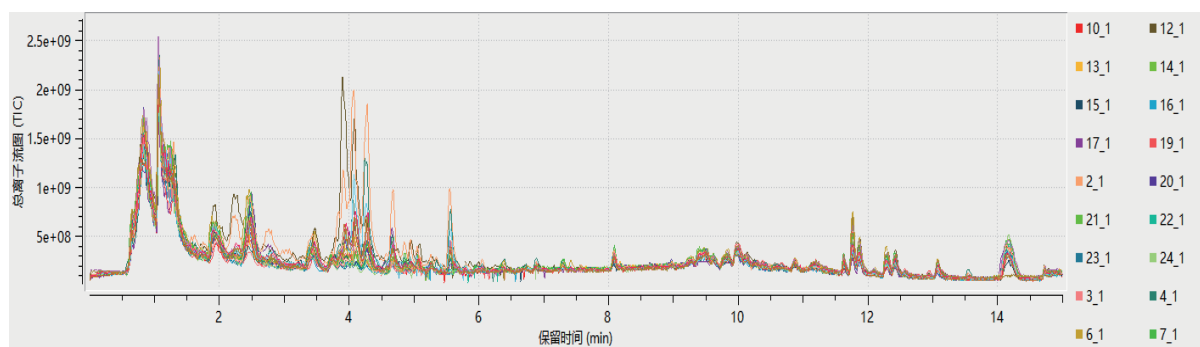

Supplement: Supplementary file 1 [file ijms-27-04399-s001.zip › supplementary material/Fig. S2.pdf]
